# The adaptive regulation of thiamine pyrophosphokinase-1 facilitates malignant growth during supplemental thiamine conditions

**DOI:** 10.18632/oncotarget.26259

**Published:** 2018-10-23

**Authors:** Hunter C. Jonus, Bradley S. Hanberry, Shivani Khatu, Jaeah Kim, Hendrik Luesch, Long H. Dang, Michael G. Bartlett, Jason A. Zastre

**Affiliations:** ^1^ Department of Pharmaceutical and Biomedical Sciences, College of Pharmacy, University of Georgia, Athens, GA, United States of America; ^2^ Department of Pediatrics, Emory University, Atlanta, GA, United States of America; ^3^ Department of Medicinal Chemistry, College of Pharmacy, University of Florida, Gainesville, FL, United States of America; ^4^ Division of Hematology/Oncology, Department of Internal Medicine, University of Florida Shands Cancer Center, University of Florida, Gainesville, FL, United States of America

**Keywords:** thiamine, cancer, hypoxia-inducible factor-1α, reactive oxygen species, thiamine pyrophosphokinase-1

## Abstract

Supplemental levels of vitamin B1 (thiamine) have been implicated in tumor progression. Tumor cells adaptively up-regulate thiamine transport during hypoxic stress. Upon uptake, thiamine pyrophosphokinase-1 (TPK1) facilitates the rapid phosphorylation of thiamine into thiamine pyrophosphate (TPP). However, the regulation of TPK1 during hypoxic stress is undefined. Understanding how thiamine homeostasis changes during hypoxia will provide critical insight into the malignant advantage supplemental thiamine may provide cancer cells. Using Western blot analysis and RT-PCR, we have demonstrated the post-transcriptional up-regulation of TPK1 in cancer cells following hypoxic exposure. TPK1 expression was also adaptively up-regulated following alterations of redox status by chemotherapeutic and antioxidant treatments. Although TPK1 was functionally up-regulated by hypoxia, HPLC analysis revealed a reduction in intracellular TPP levels. This loss was reversed by treatment with cell-permeable antioxidants and corresponded with reduced ROS production and enhanced cellular proliferation during supplemental thiamine conditions. siRNA-mediated knockdown of TPK1 directly enhanced basal ROS levels and reduced tumor cell proliferation. These findings suggest that the adaptive regulation of TPK1 may be an essential component in the cellular response to oxidative stress, and that during supplemental thiamine conditions its expression may be exploited by tumor cells for a redox advantage contributing to tumor progression.

## INTRODUCTION

Malignant cells experience elevated levels of reactive oxygen species (ROS) during tumor progression. This multifactorial byproduct arises from events including enhanced metabolic activity, increased activity of ROS-producing enzymes, dysfunctional mitochondrial metabolism, activated immune responses, intratumoral hypoxia, and therapeutic intervention (i.e. chemotherapeutics, ionizing radiation) [1, 2]. Moderate increases in ROS are tumorigenic as oxygen radicals provide critical secondary messengers in oncogenic signaling cascades and promote genomic instability. However, a delicate balance of ROS must be maintained as excessive levels cause damage to DNA, proteins, and lipids resulting in cellular senescence and apoptosis [1]. During tumor initiation and progression, antioxidant pathways are up-regulated aiding to limit the damaging effects of ROS [2]. For example, constitutive activation of the transcription factor Nuclear factor erythroid-2–related factor 2 (NFE2L2, NRF2) in tumor cells up-regulates levels of the endogenous glutathione machinery, promoting tumorigenicity [3]. In hypoxic tumor microenvironments, the stabilization of the oncogenic transcription factor hypoxia-inducible factor-1α (HIF1A, HIF-1α) regulates the expression of pyruvate dehydrogenase kinase-1 (PDK1), which subsequently limits ROS production by phosphorylating pyruvate dehydrogenase (PDH) and restricting mitochondrial metabolism [4]. siRNA-mediated silencing of HIF-1α increases intracellular ROS levels both *in vitro* and *in vivo*, highlighting HIF-1α’s critical role in ROS maintenance [5].

Tumor cells may also exploit dietary antioxidants as an alternative means to balance intracellular redox status [2]. Dietary antioxidants, which consist of a broad range of molecular classes including polyphenols, carotenoids, and tocopherols, commonly exist in the form of vitamins [6]. Vitamin E and its cell-permeable mimetic Trolox have been demonstrated to accelerate tumor progression *in vivo* and enhance the migrative and invasive properties of tumor cells *in vitro* [7, 8]. Supplemental vitamin E also protects against protein oxidation during hypoxia and hypoglycemia induced oxidative stress [9]. Vitamin B1 (thiamine) and its activated cofactor form, thiamine pyrophosphate (diphosphate; TPP) have also exhibited antioxidant activity and can suppress the generation of superoxide, hydroperoxide, and hydroxyl radicals [10]. Supplemental doses of thiamine can promote the *in vivo* growth of malignant tumors [11, 12]. The uptake of vitamin B1, or thiamine, was recently demonstrated to be adaptively up-regulated in tumor cells during hypoxic stress, but it remains unclear how increasing intracellular thiamine could be advantageous to hypoxic tumor cells [13].

As an essential micronutrient, thiamine must be obtained from the diet to maintain metabolism in all cells. The Solute Carrier (SLC) transporters THTR1 (*SLC19A2*) and THTR2 (*SLC19A3*) facilitate the absorption and cellular uptake of thiamine from the plasma [14]. Thiamine pyrophosphokinase-1 (TPK1) subsequently phosphorylates thiamine into TPP [15]. The resulting TPP moiety canonically functions as a required cofactor for multiple enzymes in the metabolic network including PDH, α-ketoglutarate dehydrogenase (OGDH), and transketolase (TKT). Of these, up-regulation of TKT occurs in cancerous tissue and promotes tumor progression [16, 17]. TKT links glycolysis with the pentose phosphate pathway (PPP) by catalyzing a reversible reaction that produces ribose-5-phosphate for use in nucleotide synthesis essential to tumor cell proliferation. Boros *et al.* found that malignant cells generate 85% of their necessary ribose through the non-oxidative portion of the PPP [18]. The activity of TKT within the PPP also facilitates the maintenance of NADPH pools and balance of the cellular redox status [16]. Though the functionality remains unresolved, TKT expression has been shown to increase ∼15-fold in hypoxia [19]. Therefore, increasing thiamine supply during hypoxia may support TKT activity in a canonical cofactor fashion. Alternatively, thiamine as well as TPP may serve other non-canonical functions during hypoxic stress potentially as antioxidants.

We have previously established an increase in the expression of *SLC19A2* and *TPK1* in breast cancer tissue when compared to normal breast tissue [20]. Furthermore, HIF-1α directly transactivates the adaptive expression of *SLC19A3* and enhances thiamine uptake during hypoxic stress [13, 21]. Despite thiamine’s implicit requirement for cellular metabolism within hypoxic tumor microenvironments, how changes in thiamine homeostasis impact malignant progression remain unclear. Tiwana *et al.* recently demonstrated TPK1, the enzyme responsible for the production of TPP, as a critical component of tumor cell survival following exposure to ionizing radiation [22]. Unfortunately, there exists limited knowledge regarding the regulation of TPK1 in cancer cells and how thiamine supplementation functions to enhance malignant progression.

## RESULTS

### Induction of TPK1 protein during hypoxia correlates with HIF-1α

**Figure 1:**
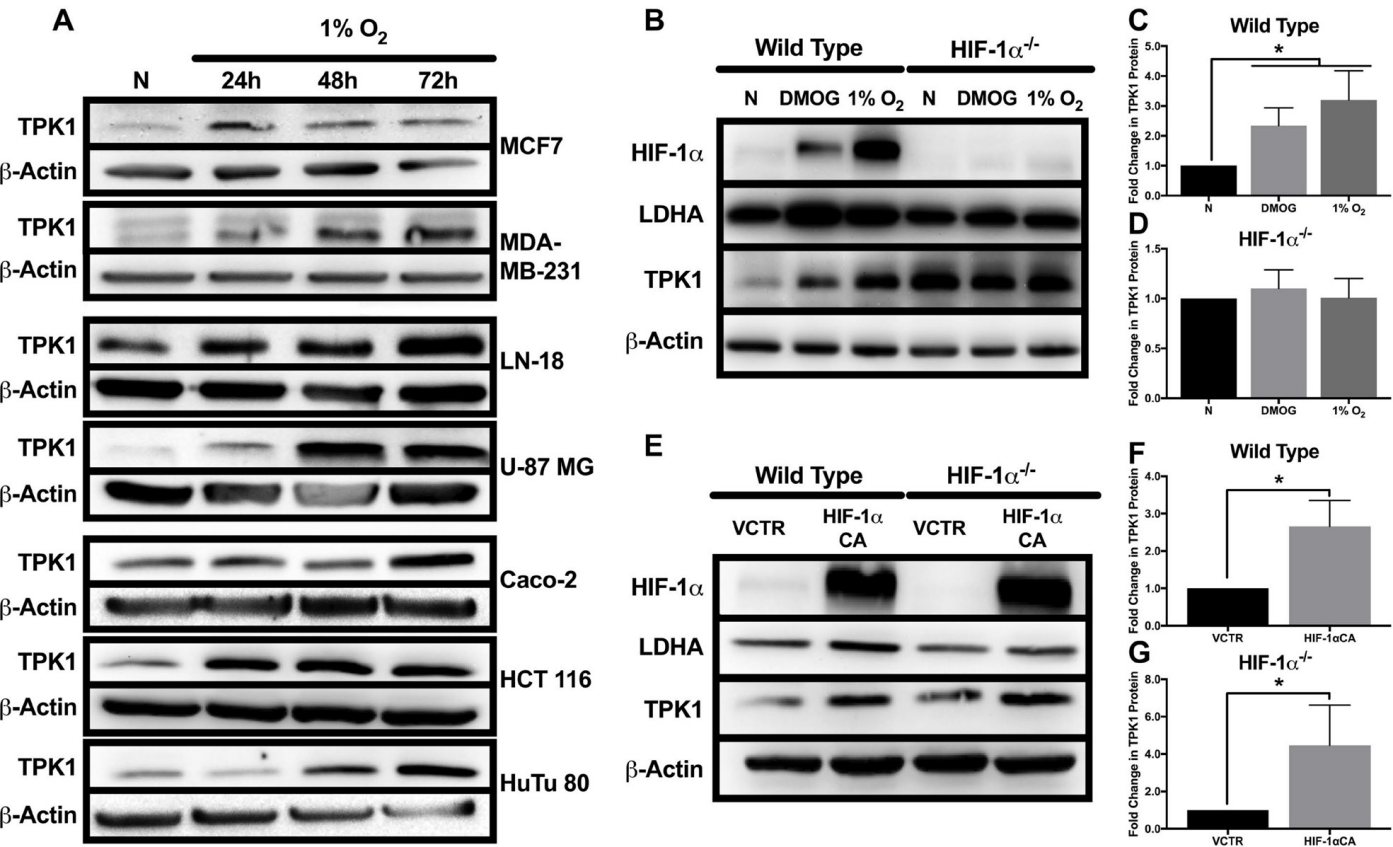
Effect of hypoxic stress and HIF-1α on TPK1 expression (**A**) Representative Western blots demonstrating TPK1 protein expression in WCLs isolated from seven tumor cell lines with tissue origins including breast (MCF7, MDA-MB-231), brain (LN-18, U-87 MG), and intestine (Caco-2, HCT 116, HuTu 80) following treatment with 1% O_2_ for 24, 48, and 72 h relative to normoxic control (N). β-Actin expression serves as the loading control. (**B**) Representative Western blots demonstrating HIF-1α, LDHA, and TPK1 protein expression in WCLs isolated from wild type and HIF-1α^–/–^ HCT 116 cells seeded at 1250 cells/cm^2^ and treated with 150 μM DMOG or 1% O_2_ for 24 h relative to normoxic control (N). (**C**, **D**) Densitometry analysis of the fold change in TPK1 expression ± standard deviation (SD) following DMOG and 1% O_2_ treatment in wildtype and HIF-1α^–/–^ HCT 116 cells compared to normoxic control (N) including *n* = 4 independent experiments for wild type and *n* = 3 independent experiments in HIF-1α^–/–^ cells. (**E**) Representative Western blots demonstrating HIF-1α, LDHA, and TPK1 protein expression in WCLs isolated from wild type and HIF-1α^–/–^ HCT 116 cells seeded at 2500 cells/cm^2^ and transfected with 2.5 μg of HIF-1α CA plasmid DNA relative to vector control (VCTR) for 72 h. (**F**, **G**) Densitometry analysis of the fold change in TPK1 expression ± SD following HIF-1α CA overexpression in wildtype and HIF-1α^–/–^ HCT 116 cells compared to vector control including *n* = 3 independent experiments. (*) Represents statistically significant difference (*p* < 0.05) based on results of (**C**, **D**) one-way ANOVA with Tukey’s post-hoc test or (**F**, **G**) unpaired student’s *t*-test.

TPK1 expression increased following 24, 48, and 72 h exposure to 1% O_2_ in an array of cancer cell lines from multiple tissue origins including breast (MCF7, MDA-MB-231), brain (LN 18, U-87 MG), and intestine (Caco-2, HCT 116, HuTu 80) (Figure 1A). To establish the role of HIF-1α in the regulation of TPK1, we utilized HCT 116 cells since an isogenic HIF-1α^–/–^ knockout was previously developed in this cell line. Wild type and HIF-1α^–/–^ HCT 116 cells were exposed to either 1% O_2_ or the prolyl hydroxylase inhibitor DMOG for 24 h. In wild type cells, DMOG and 1% O_2_ resulted in the stabilization of HIF-1α and the ∼2 and 3-fold induction of TPK1, respectively (Figure 1B and 1C). DMOG and 1% O_2_ treatment also resulted in the induction of LDHA protein expression in wild type cells, confirming the transcriptional functionality of HIF-1α (Figure 1B). In contrast to wild type, HIF-1α^–/–^ cells demonstrated no induction of TPK1 or LDHA protein following treatment with DMOG or 1% O_2_ (Figure 1B and 1D).

To further confirm a role for HIF-1α in mediating TPK1 expression, we utilized a constitutively active form of HIF-1α (HIF-1α CA) kindly provided by Dr. Hayakawa [23]. Wild type cells transfected with HIF-1α CA demonstrated an ∼2-fold increase in TPK1 expression (Figure 1E and 1F). Transfection of HIF-1α^–/–^ cells with the HIF-1α CA resulted in an ∼4-fold enhancement in TPK1 protein expression (Figure 1E and 1G).

### Pharmacological inhibition of HIF-1α and reoxygenation attenuates TPK1 up-regulation during hypoxia

**Figure 2:**
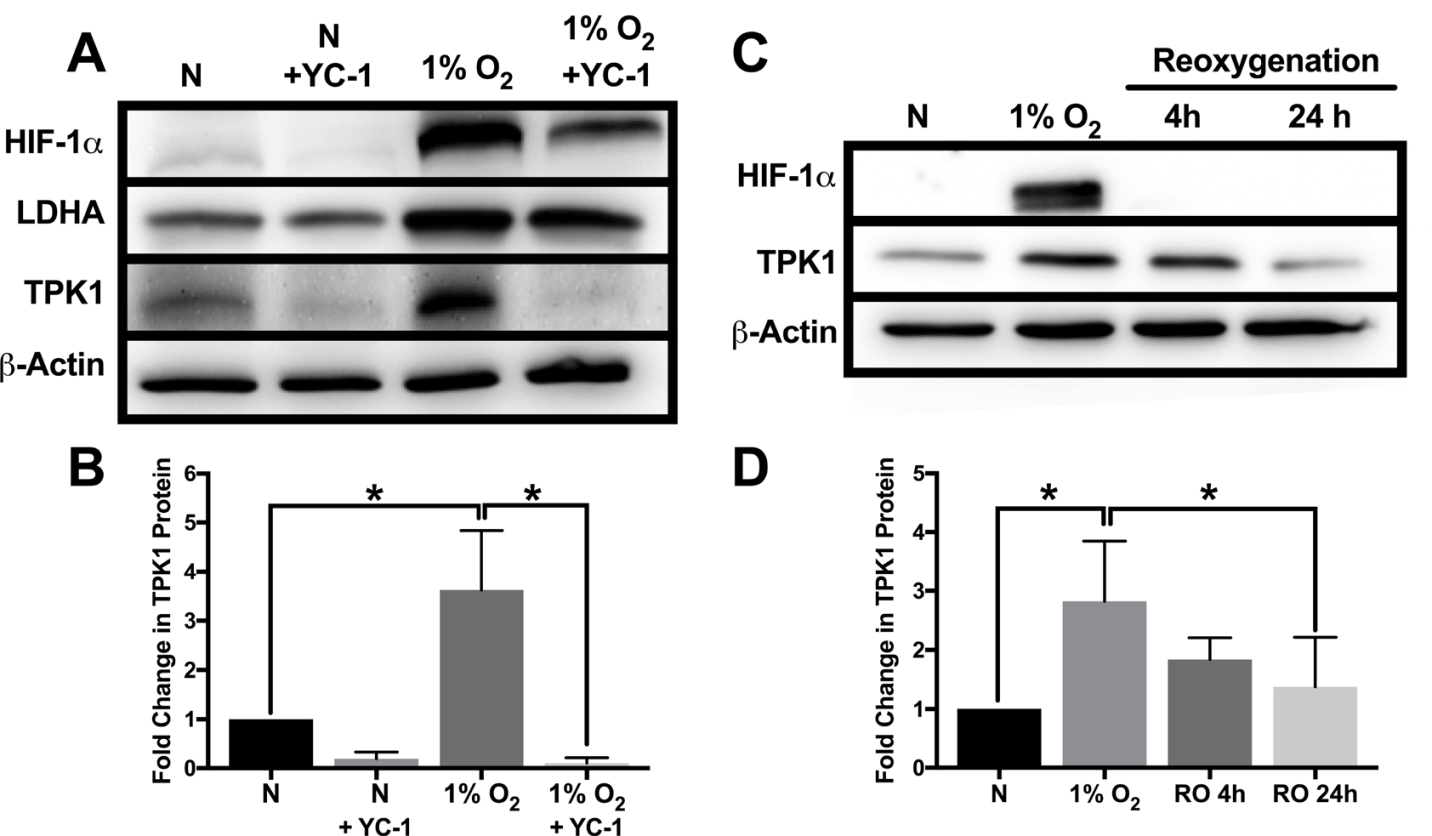
Attenuation of TPK1 expression using pharmacological inhibition of HIF-1α and reoxygenation (**A**) Representative Western blots demonstrating HIF-1α, LDHA, and TPK1 protein expression under normoxic (N) and hypoxic conditions ± YC-1 in WCLs isolated from wild type HCT 116 cells seeded at 2500 cells/cm^2^ and pre-treated with 5 μM YC-1 for 24 h prior to 48 h hypoxic exposure in the presence of 5 μM YC-1. β-Actin expression serves as the loading control. (**B**) Densitometry analysis of the fold change in TPK1 expression ± SD following exposure to 1% O_2_ in the presence or absence of YC-1 for wildtype HCT 116 cells compared to untreated normoxic control (N) including *n* = 3 independent experiments. (**C**) Representative Western blots demonstrating HIF-1α and TPK1 in WCLs isolated from wild type HCT 116 cells seeded at 1250 cells/cm^2^ and treated in 1% O_2_ for 48 h with subsequent reoxygenation at 21% O_2_ for 4 and 24 h. (**D**) Densitometry analysis of the fold change in TPK1 expression ± SD following exposure to 1% O_2_ and subsequent reoxygenation in wildtype HCT 116 cells compared to untreated normoxic control (N) including *n* = 4 independent experiments. (*) Represents statistically significant difference (*p* < 0.05) based on results of one-way ANOVA with Tukey’s post-hoc test.

To establish the effects of HIF-1α inhibition on TPK1 expression in hypoxia, we employed YC-1 as a pharmacological means to reduce both HIF-1α protein stabilization and its functional activity [24]. Treatment of HCT 116 cells with YC-1 resulted in the reduced expression of HIF-1α and its target gene LDHA in hypoxia (Figure 2A). YC-1 treatment also decreased basal expression of HIF-1α during normal oxygen conditions (Figure 2A). Inhibition of HIF-1α activity by YC-1 significantly attenuated the up-regulation of TPK1 during hypoxic stress (Figure 2A and 2B).

To determine the dynamics of TPK1 regulation following hypoxic stress, HCT 116 cells were re-oxygenated (21% O_2_) after 48 h of 1% O_2_ exposure. We observed a loss of HIF-1α and a trending reduction of TPK1 protein expression towards basal levels within 4 h of reoxygenation (Figure 2C and 2D). After 24 h of reoxygenation, restoration of TPK1 expression back to the basal normoxic level was achieved (Figure 2C and 2D).

### Increased TPK1 expression during hypoxic stress lacks transcriptional induction

**Figure 3:**
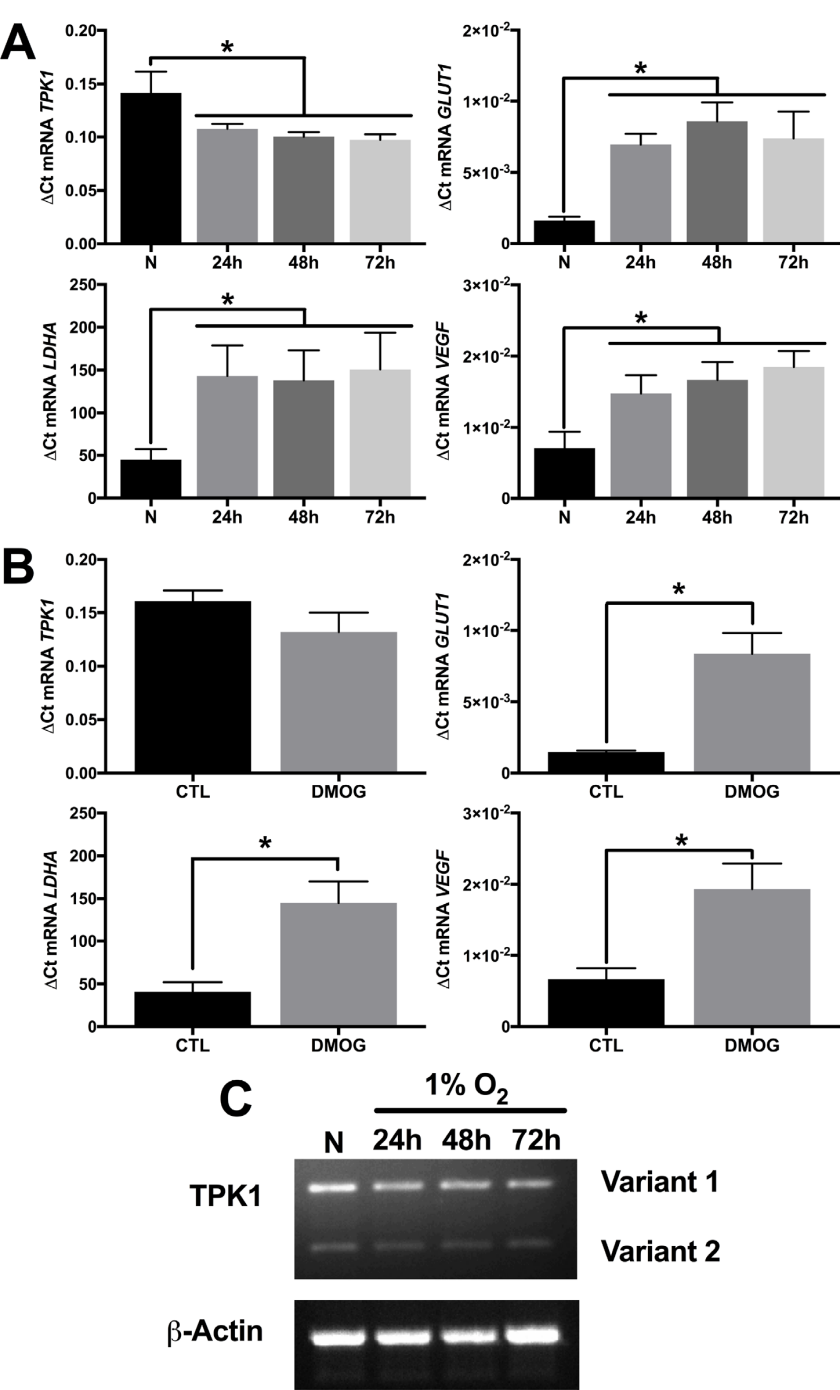
Increase in TPK1 protein expression lacks transcriptional involvement (**A**) Relative mRNA expression levels of *TPK1*, *SLC2A1*, *LDHA*, and *VEGF* determined by qRT-PCR analysis in wild type HCT 116 seeded at 1250 cells/cm^2^ and treated in 1% O_2_ for 24, 48, and 72 h relative to normoxic control (N) representative of *n* = 4 independent experiments normalized by the 2^-ΔCt^ method. (**B**) Relative mRNA expression levels of *TPK1*, *SLC2A1*, *LDHA*, and *VEGF* determined by qRT-PCR analysis in wild type HCT 116 cells seeded at 1250 cells/cm^2^ and treated with 150 μM DMOG for 24 h relative to untreated control (CTL) representative of *n* = 3 independent experiments normalized by the 2^-ΔCt^ method. (**C**) Qualitative *TPK1* splice variant expression (1 and 2) in wild type HCT 116 cells during normoxic (*N*) and 24, 48, and 72 h 1% O_2_ exposure. *β-Actin* expression serves as the loading control. (*) Represents statistically significant difference (*p* < 0.05) based on results of (**A**) one-way ANOVA with Tukey’s post-hoc test or (**B**) unpaired student’s *t*-test.

In contrast to the increase in TPK1 protein during hypoxic stress, no increase in mRNA expression of *TPK1* (combined variant 1 and 2) was observed after 24, 48, or 72 h of 1% O_2_ treatment in HCT 116 cells (Figure 3A). Instead, these treatments resulted in a slight, but significant decrease in *TPK1* transcript expression (Figure 3A). Consistent with the hypoxic driven transcriptional activity of HIF-1α, a significant increase in the mRNA of three well-defined gene targets, including *SLC2A1*, *LDHA*, and *VEGF* was observed (Figure 3A). Treatment of HCT 116 cells with the hypoxia mimetic DMOG for 24 h demonstrated a similar lack of mRNA induction for *TPK1* and a significant increase in the transcription of the HIF-1α target genes *SLC2A1*, *LDHA*, and *VEGF* (Figure 3B). Since our primers designed for the quantitative detection of TPK1 gene expression simultaneously detect both splice variant 1 and 2, we developed alternative primers to independently analyze the expression of both variants. Qualitative analysis of the individual expression of both *TPK1* genetic splice variants in HCT 116 cells further confirmed that neither variant 1 nor variant 2 was up-regulated during hypoxic exposure up to 72 h (Figure 3C).

### HIF-1α independent induction of TPK1 involves ROS

**Figure 4:**
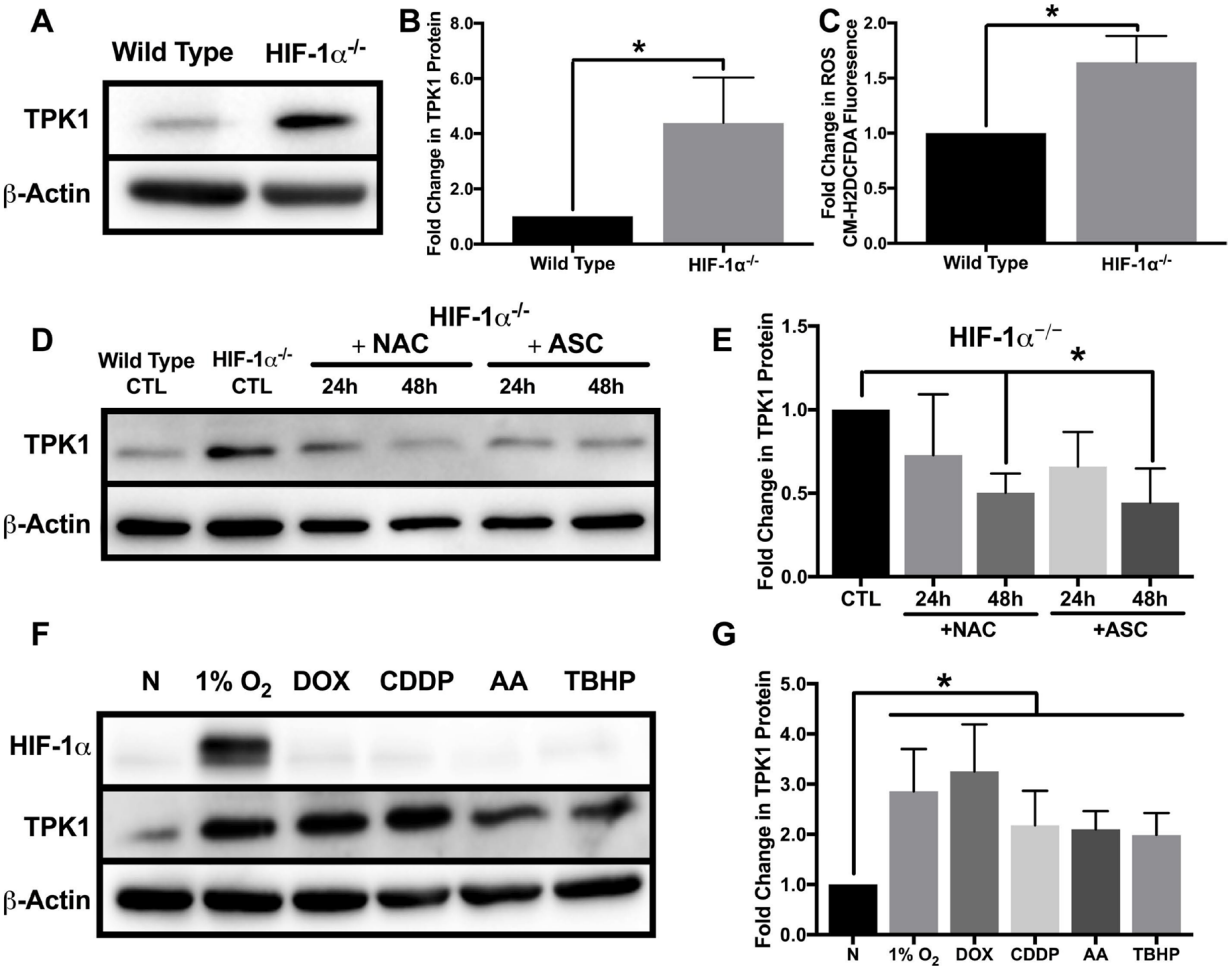
Oxidative stress mediated regulation of TPK1 (**A**) Representative Western blot demonstrating TPK1 expression in WCLs isolated from isogenic wild type and HIF-1α^–/–^ HCT 116 cells. β-Actin expression serves as the loading control. (**B**) Densitometry analysis of the fold change in TPK1 expression ± SD for HIF-1α^–/–^ HCT 116 compared to wild type HCT 116 cells including *n* = 5 independent experiments. (**C**) Fold change in CM-H2DCFDA median fluorescence intensity ± SD demonstrating fold change in ROS levels between HIF-1α^–/–^ relative to wild type HCT 116 cells including *n* = 4 independent experiments. (**D**) Representative Western blot demonstrating TPK1 expression in WCLs isolated from HIF-1α^–/–^ HCT 116 cells seeded at 1250 cells/cm^2^ and treated with 1 mM NAC or 100 μM ASC for 24 and 48 h relative to untreated HIF-1α^–/–^ HCT 116 CTL (expression of TPK1 in wild type cells provided for comparison). (**E**) Densitometry analysis of the fold change in TPK1 expression ± SD for HIF-1α^–/–^ HCT 116 cells treated with NAC and ASC compared to untreated HIF-1α^–/–^ HCT 116 control including *n* = 4 independent experiments. (**F**) Representative Western blots demonstrating HIF-1α and TPK1 protein expression in WCLs isolated from wild type HCT 116 cells seeded at 1250 cells/cm^2^ and treated with 1% O_2_, 0.1 μM DOX, 10 μM CDDP, 5 μM AA or 10 μM TBHP for 24 h relative to normoxic control (N). (**G**) Densitometry analysis of the fold change in TPK1 expression ± SD for wild type HCT 116 cells treated with 1% O_2_, DOX, CDDP, AA or TBHP compared to untreated normoxic control (N) including *n* = 6 independent experiments. (*) Represents statistically significant difference (*p* < 0.05) using (**B**, **C**) an unpaired student’s *t*-test or (**E**, **G**) one-way ANOVA with Tukey’s post-hoc test.

While establishing the differential regulation of TPK1 in wild type and HIF-1α^–/–^ HCT 116 cells, we noted a significant difference in the basal level of TPK1 protein between these two isogenic cell lines (Figure 1B and 4A). HIF-1α^–/–^ HCT 116 cells exhibited an ∼4-fold increase in TPK1 protein compared to wild type (Figure 4B). Despite significant up-regulation of TPK1 protein following HIF-1α knockout, we found no change in TPK1 mRNA expression between wild type and HIF-1α^–/–^ HCT 116 cells (Supplementary Figure 1). It was previously reported that the genetic knockdown of HIF-1α results in increased levels of ROS [5]. Consistent with these findings, we demonstrate that the knockout of HIF-1α in HCT 116 cells resulted in an ∼1.5-fold increase in ROS (Figure 4C).

To determine the potential influence of ROS on TPK1 expression, we cultured HIF-1α^–/–^ HCT 116 cells with the antioxidants N-acetylcysteine (NAC) or ascorbate (ASC) for 24 and 48 h. NAC and ASC supplementation have both been previously demonstrated to reduce tumor cell ROS levels *in vitro* [25, 26]. Following 48 h of treatment, both NAC and ASC supplementation significantly reduced basal TPK1 expression in HIF-1α^–/–^ cells to comparable levels observed in wild type cells (Figure 4D and 4E). Since antioxidant treatment of HIF-1α^–/–^ HCT 116 reduced the level of TPK1 protein, we tested whether the induction of ROS in wild type cells would induce TPK1 expression. Wild type HCT 116 cells were treated with two chemotherapeutics, doxorubicin (DOX) and cisplatin (CDDP), previously demonstrated to generate ROS [27, 28]. Cells were also treated with antimycin a (AA), which inhibits complex III of the electron transport chain to elevate ROS and the oxidant *tert*-butyl hydroperoxide (TBHP) for 24 h. Each treatment significantly induced the expression of TPK1 compared to control (Figure 4F and 4G).

### Thiamine pyrophosphate production and consumption supports antioxidant function

**Figure 5:**
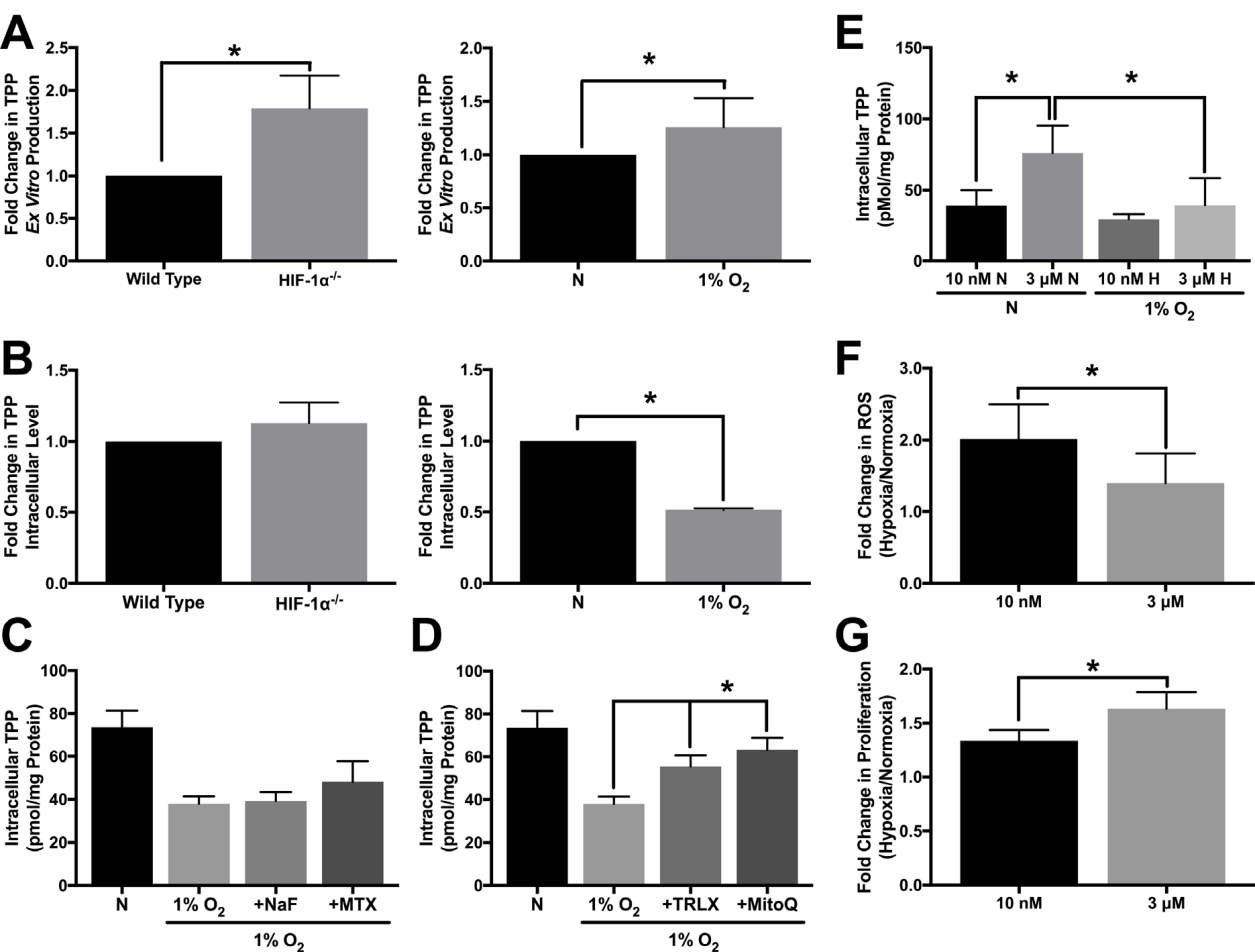
Altered TPP homeostasis during hypoxic stress (**A**) HPLC analysis demonstrating *ex vitro* TPP production as fold change in TPP ± SD comparing wild type and HIF-1α^–/–^ HCT 116 cells seeded at 1250 cells/cm^2^ and cultured for 96 h or wild type cells seeded at 1250 cells/cm^2^ and treated with 1% O_2_ for 24 h relative to normoxic control (N) including *n* = 5 independent experiments. (**B**) HPLC analysis demonstrating intracellular TPP levels as fold change in TPP ± SD comparing wild type and HIF-1α^–/–^ HCT 116 cells seeded at 1250 cells/cm^2^ and cultured for 96 h or wild type cells seeded at 1250 cells/cm^2^ and treated with 1% O_2_ for 24 h relative to normoxic control (N) including *n* = 3 independent experiments. (**C**, **D**) HPLC analysis demonstrating mean intracellular TPP levels ± SD established in wild type HCT 116 cells seeded at 25,000 cells/cm^2^ and pretreated with 500 μM NaF, 100 μM MTX, 500 μM TRLX or 10 μM MitoQ for 12 h prior to hypoxic exposure for 24 h with sustained exposure to each compound including *n* = 5 independent experiments. (**E**) HPLC analysis demonstrating mean intracellular TPP levels ± SD established in wild type HCT 116 cells exposed to 10 nM or 3 μM thiamine for 5 d prior to seeding at 50,000 cells/cm^2^ and exposure to 1% O_2_ for 24 h including *n* = 3 independent experiments for 10 nM and *n* = 7 independent experiments for 3 μM. (**F**) Effect of thiamine dose on hypoxia-induced ROS levels demonstrated by hypoxic to normoxic fold change in MitoSOX median fluorescence intensity ± SD in wild type HCT 116 cultured in 10 nM or 3 μM thiamine for 5 d prior to seeding at 50,000 cells/cm^2^ and exposure to 1% O_2_ for 48 h including *n* = 3 independent experiments. (**G**) Effect of thiamine dose on hypoxic tumor cell proliferation demonstrated by hypoxic to normoxic fold change in live cell count ± SD determined by trypan blue exclusion. Wild type HCT 116 cells were seeded at 500 cells/cm^2^ were grown for 5 d in either 1% O_2_ or normoxia including *n* = 5 independent experiments. (*) Represents statistically significant difference (*p* < 0.05) based on results of (**A**, **B**, **F**, **G**) an unpaired student’s *t*-test or (**C**–**E**) one-way ANOVA with Tukey’s post-hoc test.

To test the functionality of TPK1 in HCT 116 cells after hypoxic treatment and in HIF-1α^–/–^ cells, we used an *ex vitro* functionality assay. As a positive control that the assay system was capable of producing TPP, we overexpressed TPK1 in HCT 116 cells. TPK1 overexpression resulted in an ∼200-fold increase in the amount of TPP produced compared to control cells (Supplementary Figure 2). A significant increase in TPP production in HIF-1α^–/–^ cells relative to wild type as well as in hypoxia treated HCT 116 wild type cells relative to normoxic control was observed confirming the functional increase in TPK1 expression (Figure 5A). Although HIF-1α^–/–^ HCT 116 cells demonstrated a greater ability to produce TPP *ex vitro* relative to wild type cells, no significant differences in the intracellular levels of thiamine or TPP between the two cell lines were detected (Figure 5B, Supplementary Figure 3). However, significant decreases in thiamine and TPP levels were observed in HCT 116 cells treated with 1% O_2_ for 24 h compared to normoxic conditions (Figure 5B, Supplementary Figure 4A).

To assess changes in TPP during hypoxic stress, HCT 116 cells were treated with sodium fluoride (NaF) and methotrexate (MTX). As a cell permeable phosphatase inhibitor, NaF was used to limit dephosporylation of TPP to either thiamine monophosphate (TMP) or thiamine. MTX was used as a recognized inhibitor of the Reduced Folate Carrier (*SLC19A1*) transporter, which was previously demonstrated to function in the extracellular transport of TPP [29]. Neither of these treatments significantly preserved or accumulated thiamine, TMP, or TPP levels in hypoxic HCT 116 cells (Figure 5C, Supplementary Figure 4A, 4B). Alternatively, we considered that TPP may be consumed as an intracellular antioxidant. Figure 5D demonstrates that treatment with two different cell permeable antioxidants trolox (TRLX) and MitoQuinone (MitoQ), resulted in a significant preservation of TPP during hypoxia in HCT 116 cells. TRLX treatment also significantly increased intracellular thiamine concentration under the same conditions (Supplementary Figure 4A).

Considering intracellular TPP concentrations may be highly dynamic, HCT 116 cells were next cultured in a more physiologically relevant concentration of thiamine (10 nM) to assess if the dose of thiamine has any effect on TPP homeostasis. Culturing cells in the supplemental 3 μM dose of thiamine lead to significantly higher intracellular thiamine and TPP levels under normoxic conditions compared to cells cultured with 10 nM thiamine (Figure 5E, Supplementary Figure 5). The exposure of cells cultured in 10 nM thiamine to 1% O_2_ demonstrated no significant difference in intracellular TPP levels compared to those cultured in normoxia (Figure 5E). However, a significant loss in TPP was observed for cells cultured with 3 μM thiamine in 1% O_2_ compared to normoxic conditions (Figure 5E). We next questioned whether altering the intracellular level of TPP through supplemental thiamine influences hypoxia induced-ROS levels. When HCT 116 cells were placed in 1% O_2_ for 48 h, there was a significant reduction in ROS for cells grown in the presence of 3 μM thiamine compared to 10 nM thiamine (Figure 5F). A significant increase in HCT 116 proliferation during hypoxic stress was also observed when supplemented with 3 μM thiamine compared to 10 nM thiamine (Figure 5G).

### TPK1 facilitates cellular proliferation in the presence of supplemental thiamine

**Figure 6:**
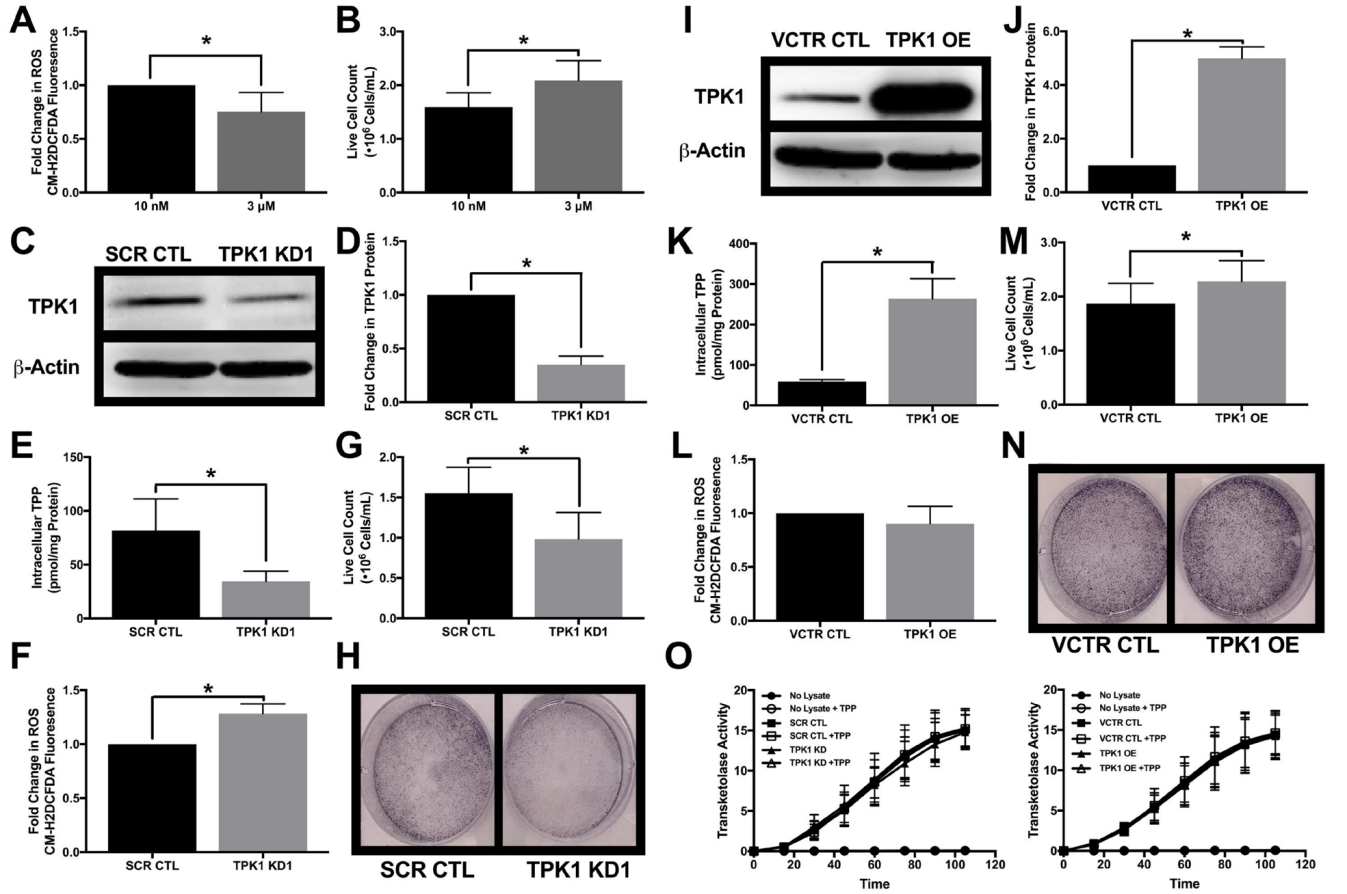
Impact of TPK1 on tumor cell proliferation during supplemental thiamine conditions (**A**) Fold change in CM-H2DCFDA median fluorescence intensity ± SD comparing fold change in ROS levels of wild type HCT 116 cells seeded at 10,000 cells/cm^2^ and supplemented with 3 μM thiamine compared to 10 nM for 5 d including *n* = 4 independent experiments. (**B**) Effect of thiamine dose on cell proliferation demonstrated by mean live cell count ± SD determined by trypan blue exclusion of wild type HCT 116 seeded at 1250 cells/cm^2^ supplemented with 3 μM thiamine or 10 nM for 5 d including *n* = 5 independent experiments. (**C**) Representative Western blot demonstrating TPK1 expression in WCLs isolated from wild type HCT 116 cells transfected with non-silencing scramble control (SCR CTL) or TPK1 targeted (TPK1 KD1) siRNA for 72 h. β-Actin expression serves as the loading control. (**D**) Densitometry analysis of the fold change in TPK1 expression ± SD for wild type HCT 116 cells transfected with TPK1 KD1 siRNA compared to SCR CTL including *n* = 3 independent experiments. (**E**) HPLC analysis demonstrating mean intracellular TPP levels ± SD established in wild type HCT 116 cells transfected with SCR CTL or TPK1 KD1 siRNA for 72 h including *n* = 5 independent experiments. (**F**) Fold change in CM-H2DCFDA median fluorescence intensity ± SD comparing fold change in ROS levels of wild type HCT 116 cells transfected with TPK1 KD1 compared to SCR CTL siRNA for 96 h including *n* = 6 independent experiments. (**G**) Effect of TPK1 knockdown on tumor cell proliferation demonstrated by mean live cell count ± SD determined by trypan blue exclusion of wild type HCT 116 cells transfected with SCR CTL or TPK1 KD1 siRNA for 96 h including *n* = 5 independent experiments. (**H**) Images of formalin fixed cells stained with crystal violet following transfection with SCR CTL or TPK1 KD1 siRNA for 96 h. (**I**) Representative Western blot demonstrating TPK1 expression in WCLs isolated from wild type HCT 116 cells transfected with pcDNA3.1+ vector control (VCTR CTL) or pcDNA-*TPK1* (TPK1 OE) vectors for 72 h. (**J**) Densitometry analysis of the fold change in TPK1 expression ± SD for wild type HCT 116 cells transfected with TPK1 OE compared to VCTR CTL including *n* = 3 independent experiments. (**K**) HPLC analysis demonstrating mean intracellular TPP levels ± SD established in wild type HCT 116 cells transfected with VCTR CTL or TPK1 OE vectors for 72 h including *n* = 3 independent experiments. (**L**) Fold change in CM-H2DCFDA median fluorescence intensity ± SD comparing fold change in ROS levels of wild type HCT 116 cells transfected with TPK1 OE compared to VCTR CTL for 96 h including *n* = 3 independent experiments. (**M**) Effect of TPK1 overexpression on tumor cell proliferation demonstrated by mean live cell count ± SD determined by trypan blue exclusion of wild type HCT 116 cells transfected with VCTR CTL or TPK1 OE vectors for 96 h including *n* = 5 independent experiments. (**N**) Images of formalin fixed cells stained with crystal violet following transfection with VCTR CTL or TPK1 OE vectors for 96 h. (**O**) The average rate of TKT activity quantified from the reduction of NADH over time ± the addition of exogenous TPP in lysates isolated from wild type HCT 116 cells transfected with SCR CTL and TPK1 KD1 siRNA or VCTR CTL and TPK1 OE vectors for 72 h including *n* = 3 independent experiments for both conditions. (*) Represents statistically significant difference (*p* < 0.05) based on results of an unpaired student’s *t*-test.

HCT 116 cells were also used to define the role of TPK1 expression in tumor cell proliferation with supplemental thiamine levels. Cells were cultured in either the physiological thiamine level of 10 nM or the supplemental dose of 3 μM, and under these conditions basal ROS levels decreased with 3 μM compared to 10 nM thiamine (Figure 6A). In addition, HCT 116 cells cultured in 3 μM thiamine proliferated more rapidly than those grown in 10 nM thiamine (Figure 6B). To understand how these effects may relate to TPK1 expression, a validated siRNA construct was utilized to mediate the knockdown of TPK1 in HCT 116 cells (Figure 6C and 6D). In the presence of supplemental thiamine (3 μM), TPK1 knockdown resulted in a significant reduction in the intracellular TPP level (Figure 6E), while no significant change in the intracellular thiamine pool was observed (Supplementary Figure 6A). Figure 6F demonstrates a significant increase in basal ROS in TPK1 knockdown cells compared to CTL. TPK1 knockdown also significantly reduced HCT 116 proliferation (Figure 6G and 6H). The effects of TPK1 knockdown on HCT 116 proliferation were confirmed using a second siRNA construct with an alternative target sequence to mediate knockdown (Supplementary Figure 7).

A pcDNA3.1+ vector containing *TPK1* was used to achieve TPK1 overexpression (Figure 6I and 6J). In the presence of supplemental thiamine (3 μM), exogenous TPK1 overexpression resulted in significantly greater intracellular TPP level (Figure 6K) and a significant reduction in the level of intracellular thiamine (Supplementary Figure 6B). The overexpression of TPK1 had no significant effect on ROS levels (Figure 6L). TPK1 overexpression induced a modest but significant increase in tumor cell proliferation (Figure 6M and 6N). Altering intracellular TPP levels, either due to TPK1 knockdown or overexpression, had no impact on the functional activity of the thiamine dependent enzyme TKT (Figure 6O). Furthermore, no intracellular deficiency of TPP cofactor was detected due to TPK1 knockdown or overexpression as demonstrated by a lack of TKT activity enhancement with exogenous addition of TPP to the TKT activity assay (Figure 6O).

## DISCUSSION

Recent evidence has demonstrated that thiamine supplementation supports malignant progression by increasing tumor proliferation. Using the spontaneous tumor mouse model FVB/N-Tg(MMTV-neu), Daily *et al.* found that thiamine supplementation reduced tumor latency [12]. An increase in Ehrlich ascites tumor proliferation was also observed in mice administered thiamine at 12.5 times the recommended daily allowance (RDA) [11]. Our lab has previously established that the effects of thiamine supplementation on tumor proliferation may be supported by the up-regulation of thiamine homeostasis genes. An increase in the expression of the thiamine transporter, *SLC19A2* was found in breast cancer tissue when compared to normal breast tissue [20]. Correspondingly, the level of thiamine was higher in three out of four breast cancer cell lines compared to human mammary epithelial cells. An increase in the gene expression of *TPK1* was also observed in breast tumor tissue when compared to normal breast tissue [20]. In hypoxia, an adaptive increase in the expression of the thiamine transporter *SLC19A3* and increase in thiamine transport was found in malignant cells [13]. Here, we demonstrate a similar adaptive up-regulation for the thiamine activating enzyme TPK1 during hypoxic and oxidative stress.

Supported by both gain-of-function (hypoxia, DMOG, HIF-1α CA) and loss-of-function (YC-1, HIF-1α^–/–^) studies, the activity of the oncogenic transcription factor HIF-1α appeared to be responsible for enhancing TPK1 expression during hypoxic conditions. HIF-1α also mediates *SLC19A3*′s adaptive up-regulation during hypoxia [13]. However, the regulatory pathways for the two thiamine homeostasis genes diverge in that TPK1 expression was found to be translationally enhanced, while HIF-1α directly transactivates *SLC19A3* gene expression [21]. Our findings for the inhibitory effects of YC-1 on TPK1 expression may also provide further support for the translational regulation of TPK1 expression. In this study, the intended use for YC-1 was as a HIF-1α inhibitor [24]. However, it has also been demonstrated that YC-1 treatment may downregulate cap-dependent mRNA translation through inhibiting the phosphorylation of eukaryotic translation initiation factor 4E (eIF4E)-binding protein 1 (4E-BP1) [30, 31]. Therefore, reduced TPK1 expression under hypoxic conditions following YC-1 treatment may have been due to direct inhibition of TPK1 translation instead of through HIF-1α inhibition. The observed decrease in TPK1 expression during normoxic conditions supports inhibition of TPK1 translation by YC-1 treatment.

Rapid cellular adaptations to stress are often facilitated by translationally regulated protein responses rather than transcriptionally mediated gene expression alone [32]. Translational up-regulation of TPK1 in hypoxia may suggest an immediate requirement for TPP production in response to oxidative stress. This coincides with our finding that TPK1 expression was also regulated in a HIF-independent manner related to oxidative stress. Although we did not observe HIF-1α stabilization in our ROS-inducing treatments (i.e. DOX, CDDP, AA, TBHP), convergence of the two pathways cannot be ruled out as ROS has previously been defined as a factor in both the normoxic and hypoxic stabilization of HIF-1α [33]. Identifying secondary mediators related to both HIF-1α and ROS will be critical in further elucidating TPK1’s adaptive regulation in response to malignant stress. The oncogenic microRNA miR-155 may be a candidate as it was recently demonstrated to regulate thiamine homeostasis during malignancy by mediating the transcript and protein expression of THTR1 (SLC19A2) and TPK1 [34]. In addition, miR-155 has been demonstrated to be functionally up-regulated by hypoxia and has also been shown to deregulate redox homeostasis highlighting a potential connection to ROS [35, 36]. Future work should validate the role of miR-155 in the translational regulation of TPK1 expression.

Both thiamine and TPP have been demonstrated to act as antioxidants, scavenging superoxide and hydroxyl radicals as well as peroxide molecules [10]. The transfer of 2 H^+^ + 2 e^-^ from the aminopyrimidine ring of thiamine to free radicals has been shown to drive its direct antioxidant property [37]. Our findings demonstrate that TPP may act as an intracellular antioxidant consumed during oxidative stress in malignant cells. Like TPP, thiamine levels also decreased during the ROS-associated stress of hypoxia suggesting it may also have been consumed as an antioxidant. However, TPP has previously been demonstrated to provide greater protective effect against oxidative stress-induced damage (i.e. DNA hydroxylation) compared with thiamine [38]. Therefore, the observed loss of thiamine may have been due to an increased conversion to TPP following antioxidant consumption of TPP. Further investigation regarding the kinetics for thiamine conversion into TPP during hypoxia and oxidative stress will be required to provide these mechanistic details. *In vivo* evidence supports TPP’s ability to act as an antioxidant by establishing that TPP administration protects against CDDP-induced neuro, liver, and cardiotoxicity [38–40]. Likewise, the administration of TPP prevents against oxidative damage in ischemia reperfusion induced kidney toxicity, methotrexate induced liver toxicity, and alcohol induced hepatotoxicity [41–43]. In the brain, thiamine deficiency (lack of TPP) and oxidative stress appear to coincide and potentially contribute to neurodegenerative disorders such as Alzheimer’s disease [44]. These findings suggest that the antioxidant potential of TPP may be far reaching amongst different disease pathologies and not simply limited to the malignant state.

A dichotomy presents for the effects of dietary antioxidants on tumor progression. Epidemiological studies suggest an inverse correlation between diets rich in dietary antioxidants and cancer risk, while clinical data reveals that β-carotene, vitamin A, and vitamin E may increase the risk of cancer-associated death [6, 45]. Supplementation with the antioxidants NAC and vitamin E have recently been shown to promote tumor progression and enhance metastatic potential *in vivo* [7, 8]. Here, the adaptive up-regulation of TPK1 during ROS-inducing conditions appeared to be a cellular response to maximize TPP production. With supplemental thiamine, the level of ROS was reduced and proliferation increased. Loss of TPK1 expression sensitized tumor cells to enhanced ROS-levels, while the exogenous overexpression of TPK1 produced no further reduction in basal ROS, despite significant TPP accumulation. The lack of effect for TPK1 overexpression may be contingent on supplemental thiamine, considering that with 3 μM thiamine basal ROS was already minimal compared to the more physiological relevant level of 10 nM thiamine. The minimal impact for the exogenous overexpression of TPK1 to further reduce ROS in the presence of supplemental thiamine suggests that TPP has limited capacity to reduce cellular redox status compared to other endogenous systems (i.e.glutathione, NADPH). Our findings suggest that the endogenous adaptive regulation of TPK1 in response to malignant stress facilitates the necessary TPP production for the molecule to serve as both a cofactor and intracellular antioxidant.

*Tiwana et al.* found TPK1 expression to be a significant factor in the susceptibility of cancer cells to ionizing radiation [22]. siRNA-mediated knockdown of TPK1 decreased tumor cell survival following radiation treatment. This finding was attributed to the necessity of thiamine homeostasis in maintaining TKT activity for the production of nucleotides and DNA repair following oxidative stress [22]. However, radiation sensitization due to the silencing of TPK1 could also be linked to TPP’s apparent role as an intracellular antioxidant. Using TKT activity as a probe for thiamine-dependent enzyme activity, we found the cofactor and non-cofactor roles for TPP to be mutually exclusive. No change was found for TKT activity due to an apo-holo enzyme effect despite significant losses in TPP with siRNA directed against TPK1. There was also no demonstrated “TPP effect” through the addition of TPP to assay system or enhancement of TKT activity following TPP accumulation with the exogenous overexpression of TPK1 suggesting no intracellular TPP deficiency impacting thiamine dependent enzyme activity. One factor for further consideration will be the effects of supplemental thiamine. There was no change for intracellular TPP levels detected in hypoxia compared to normoxia when cells were grown in the physiological relevant concentration of 10 nM thiamine. This may be indicative of the basal level of TPP required to maintain function of thiamine dependent enzymes. However, in the presence of supplemental thiamine there was a loss of accumulated TPP in hypoxia compared to normoxia. We suggest this corresponds with the antioxidant function of TPP. Despite the observed loss, a similar baseline level of TPP was maintained comparable to that of 10 nM conditions highlighting the minimum required level for functional enzymatic activity. These findings are indicative of disadvantageous effects for the consumption of supplemental dietary thiamine during malignancy. Furthermore, vitamin supplements often contain 1000-6000% of the RDA for thiamine and may pose significant health hazards for cancer patients [46].

**Figure 7:**
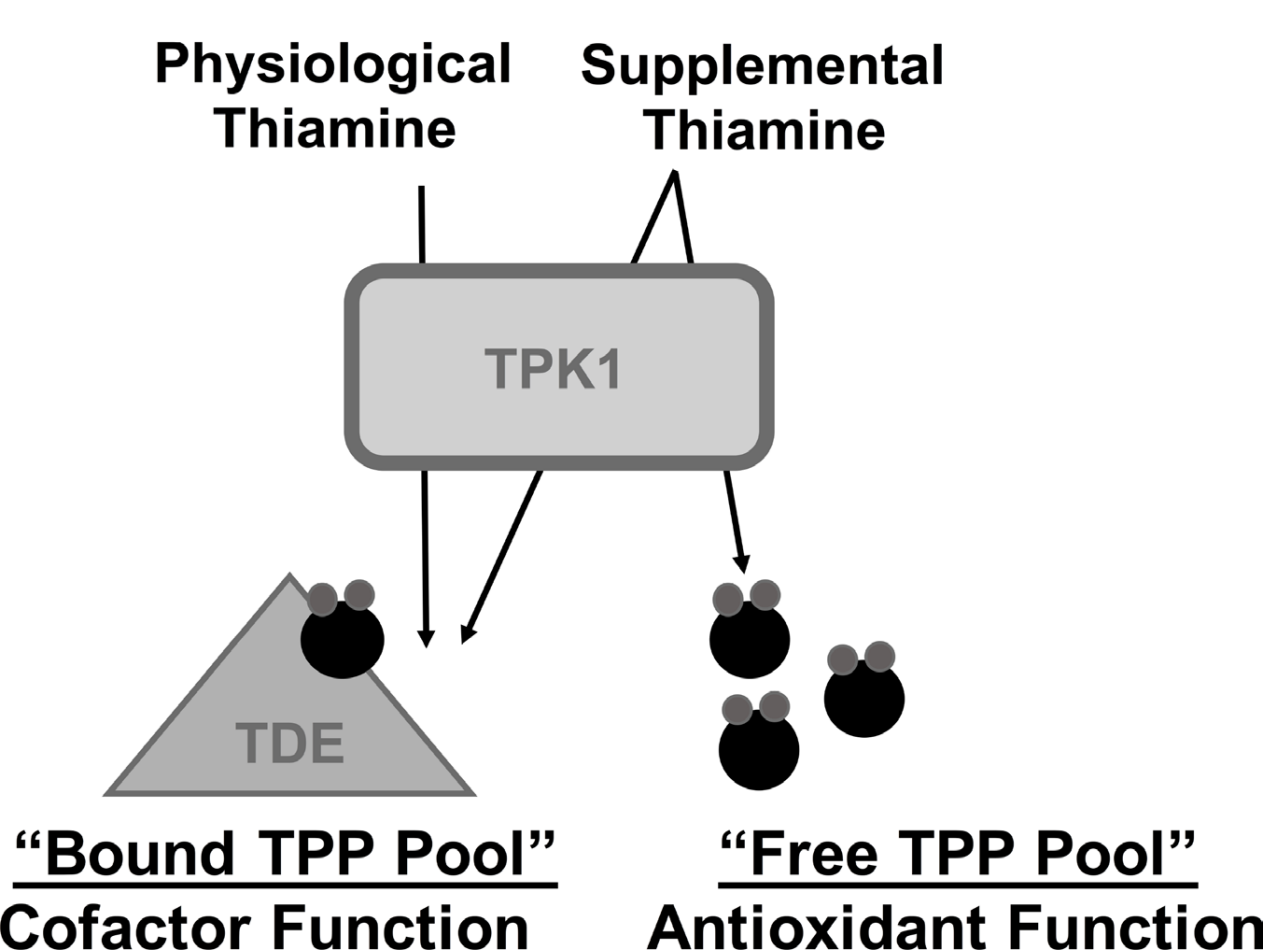
Schematic representation for the hypothesized role of TPK1 in mediating the effects of supplemental thiamine on malignant progression We hypothesize that in the presence of physiological thiamine levels, TPP produced by TPK1 maintains thiamine dependent enzyme activity (TDE) representing the bound TPP pool. However, during supplemental thiamine conditions, TPK1 up-regulation facilitates both the production of bound TPP, as well as a “free TPP pool,” which may be consumed by tumor cells through antioxidant reactions to maintain redox status and promote growth.

In conclusion, we propose that the adaptive up-regulation of TPK1 occurs during malignant stress to facilitate continuous TPP production for consumption as an intracellular antioxidant, independent of its cofactor function. This coincides with the model proposed by *Bettendorf et al.* that two independent intracellular pools of TPP exist with separate regulatory and functional fates [47]. The “cofactor” pool, which undergoes little turnover, provides the necessary amount of cofactor required to maintain the activity of thiamine dependent enzymes. The second “free” pool undergoes rapid intracellular turnover, consistent with our antioxidant consumption hypothesis (Figure 7). In response to elevated TPP turnover during stress conditions, tumor cells may exploit convergent mechanisms to rapidly maximize TPP production. First, up-regulation of thiamine transport should ensure the availability of necessary thiamine substrate to rapidly produce new TPP molecules. Supporting this proposal, thiamine transport enhances via *SLC19A3* expression during hypoxic conditions in addition to the up-regulation of thiamine homeostasis that occurs during malignancy related to *SLC19A2* expression [13, 20]. Second, our findings for the adaptive up-regulation of TPK1 during hypoxic and oxidative stress suggest an attempt to maximize intracellular conversion of thiamine to TPP. Together, the dual up-regulation of thiamine transport and TPK1 expression would serve to continuously produce TPP during malignant stress conditions. The consumption of TPP as an antioxidant may provide some insight into the numerous clinical reports of thiamine deficiency in advanced stage cancer patients [48]. It remains undefined as to why these patients become thiamine deficient, however one of the associated risk factors is the occurrence of a rapidly developing tumor [49]. Thiamine and its phosphate ester metabolites are also elevated in tumor tissue, while uninvaded control tissues show perpetual declines of the moieties throughout tumor growth [50]. Together, this may suggest that the body’s thiamine stores can be redistributed through up-regulation of thiamine homeostasis genes and exploited by tumor cells for growth and survival purposes.

## MATERIALS AND METHODS

Standard cell culture reagents including RPMI 1640 media, penicillin/streptomycin, and trypsin/EDTA were purchased from Corning (Manassas, VA). Fetal bovine serum (FBS) was obtained from Seradigm (Radnor, PA). Cell culture treated flasks and dishes were purchased from Greiner Bio-One (Monroe, NC). Chemicals including DMOG, DOX, CDDP, MTX, NAC, ASC, NaF, AA, TBHP and TRLX were purchased from Sigma Aldrich (St. Louis, MO). MitoQ was purchased from GlycoSyn Technologies (New Zealand) and YC-1 from Tocris (Minneapolis, MN).

### Cell culture

Tumor cell lines including MCF7, MDA-MB-231, LN-18, U-87 MG, Caco-2, HCT 116, and HuTu 80 were obtained from ATCC (Manassas, VA). Research Registry IDs are provided in Supplementary Table 1. HCT 116 HIF-1α^**–/–**^ cells were developed previously [51]. All cells were routinely cultured in complete RPMI 1640, which contained 10% FBS and 1% penicillin/streptomycin, at 37° C with 5% CO_2_ designated as normoxic conditions. Routine culture media also contained 0.1% Mycozap (Lonza, Verviers, Belgium) to prevent mycoplasma contamination. Wild type HCT 116 cells were confirmed to be mycoplasma free in June 2018 by IDEXX Bioresearch (Columbia, MO) Impact 1 PCR profile. Maintenance flasks of cells were grown to 60-70% confluency prior to splitting into dishes for experimental treatments. All cells used for experimentation ranged from passage 4-14.

An incubator equipped with a ProOX oxygen sensor and regulator (Biospherix, Lacona, NY) was used for hypoxia treatments. The regulator supplies nitrogen gas to maintain a designated level of 1% O_2_ within the incubator. The sensor was calibrated on a weekly basis to ensure consistent hypoxic conditions. For hypoxic treatments, culture media was replaced with complete RPMI 1640 that had been pre-equilibrated in hypoxia for a minimum of 24 h. Dishes were transferred to hypoxic incubator for the remainder of experiment and hypoxia pre-equilibrated media was changed every 24 h as necessary during extended hypoxia treatment times.

RPMI 1640 contains 3 μM thiamine, an amount approximately 300 times greater than the ∼10 nM concentration found in human serum [52]. When necessary to adjust the thiamine concentration to be consistent with physiological levels, custom formulated thiamine deficient RPMI 1640 (TD 1640) (United States Biological, Salem, MA) was utilized. TD 1640 was supplemented with 10% FBS, 1% penicillin/streptomycin, and 0.1% Mycozap to complete the medium. Supplementation of the TD 1640 medium with 10% FBS resulted in the physiologically relevant concentration of ∼ 10 nM (Supplementary Figure 8).

### Cloning and over-expression of TPK1

Although two isoforms of the *TPK1* gene exist, only variant 1 (NM_022445.3) has been shown to catalyze the activation of thiamine [15]. *TPK1* splice variant 2 (NM_001042482.1) lacks exon 7, which results in a 49 amino acid deletion in a conserved portion of the full-length protein. It is unclear if this deletion alters the functional activity for thiamine diphosphorylation [53]. *TPK1* variant 1 was cloned from human testis cDNA (Clontech Laboratories Inc., Mountain View, CA) by PCR amplification. Cloning primers for *TPK1* were constructed (F: 5′-TCC*GCTAGC*ATGGAGCATGCC-3′ and R: 5′-TCCGGTACCTTAGCTTTTGACGGCC-3′) to be flanked with *NheI* (Forward) and KpnI (Reverse) restriction sites. PCR was performed with an annealing temperature of 57° C for 32 cycles and the resulting 734 bp product was excised and purified using a Gel Extraction Kit (Omega Bio-tek, Norcross, GA). The resulting fragment was then digested and ligated into pcDNA3.1(+) (Life Technologies, Grand Island, NY). The sequence was verified using the Georgia Genomics and Bioinformatics Core (Athens, GA).

The effects of TPK1 overexpression were studied by transfecting the pcDNA3.1(+)-*TPK1* vector into HCT 116 cells. A pcDNA3.1(+) vector containing *EGFP* was used as a vector transfection control for each experiment. For ROS measurements relying on fluorescence signal, an empty pcDNA3.1(+) vector was used. Cells were seeded at 5000 cells/cm^2^ and allowed to attach for 12 h. Cells were then transfected with 17.5 ng of DNA/cm^2^ at a final concentration of 0.01% lipofectamine (Promega, Madison, WI).

### siRNA-mediated TPK1 knockdown

siRNA knockdown of TPK1 was achieved using functionally validated constructs purchased from Qiagen (Supplementary Table 2). HCT 116 cells were seeded at 5000 cells/cm^2^ and constructs targeting TPK1 or scrambled control sequences were reverse transfected using a final siRNA concentration of 20 nM with Qiagen HiPerfect transfection reagent at a ratio of 1:400 (final volume).

### Assessment of gene expression

Differential gene expression of *TPK1* splice variants was qualitatively assessed using PCR analysis. RNA was extracted using the E.Z.N.A Total RNA Kit I (Omega Bio-Tek, Norcross, GA) following the manufacturer’s provided protocol. RNA concentration was determined using a Nanodrop 2000c Spectrophotometer (Thermo Scientific, Rockford, IL). cDNA was reverse transcribed from 1 μg of isolated RNA using the qScript cDNA Synthesis Kit (Quanta BioSciences, Gaithersburg, MD). The expression of *TPK1* splice variants was analyzed by designing primers to simultaneous amplify *TPK1* variant 1 (438 bp) and variant 2 (291 bp). Primer sequences were: F 5′-CCTGAATTCATCAATGGAGACTTTG-3′ and R 5′- AGCAAGCACATCATTTGTGAGG-3′. PCR reaction was carried out using EconoTaq Plus Green 2X Master Mix (Lucigen, Middleton, WI) in a DNA Thermal Cycler (Thermo Scientific, Rockford, IL) with annealing at 57° C for 32 cycles. The resulting product was electrophoresed on a 1.5% agarose ethidium bromide gel. Fragments were visualized with ultraviolet light using a Biorad Gel Doc EZ Imager (BioRad, Hercules, CA).

The combined total expression of *TPK1* variants 1 and 2 and the HIF-1α target genes lactate dehydrogenase (*LDHA*), vascular endothelial growth factor (*VEGF*), and *SLC2A1* (GLUT1) was determined by quantitative real-time PCR using a Light-Cycler 480 II (Roche Applied Science, Indianapolis, IN). For detection, gene specific primers were designed using the Roche Universal Probe Library assay design center in correspondence with a specific Roche hydrolysis probe labeled with fluorescein (FAM). The primer/probe pairs used for this study are listed in Supplementary Table 3. Human TATA-binding protein (*TBP*) was used as a reference gene to calculate relative gene expression based on the 2^-DCt^ method with an assumed efficiency of 2.

### Evaluation of protein expression

To assess changes in TPK1 protein expression, cells were harvested as whole cell lysates (WCL) for Western blot analysis. WCLs were prepared by washing treated cells in ice-cold phosphate buffered saline (PBS) followed by immediate lysis using lysis buffer (1% Nonidet P-40 (NP40), 0.1% sodium dodecyl sulfate (SDS), 0.5% sodium deoxycholate, 0.01% sodium azide, 50 mM tris, 250 mM NaCl, and 1 mM ethylenediaminetetraacetic acid (EDTA) at pH = 8.5) supplemented with phenylmethanesulfonylfluoride (Calbiochem, La Jolla, CA) and protease/phosphatase inhibitors (G-Biosciences, St. Louis, MO). Lysates were collected and centrifuged at 17,000 × g using a Microfuge 22R Centrifuge (Beckman Coulter, Brea, CA) for 20 min at 4° C. The supernatant was collected and total protein content was determined using a BCA protein assay (Thermo Scientific, Rockford, IL). WCLs (50 μg) from each treatment were resolved by electrophoresis using a 12% SDS-PAGE gel. Separated proteins were then transferred to polyvinylidene difluoride membranes. Membranes were blocked with 5% non-fat milk in tris buffered saline-tween 20 (TBS-T) for 1 h at room temperature. Membranes were then immunoblotted with primary antibody for 12 h at 4° C. TPK1 and HIF-1α expression were assayed with β-Actin (ACTB) expression serving as loading control. The expression of LDHA was also assayed as a marker of functionally active HIF-1α [54]. Supplementary Table 4 provides all information regarding manufacturer and dilution (in TBS-T) for individual antibodies. After the primary antibody incubation, blots were washed three times with TBS-T (10 min each) and then exposed to horseradish peroxidase (HRP)-conjugated goat anti-mouse or goat anti-rabbit secondary antibody (Bethyl Laboratories, Montgomery TX) at a 1:10,000 dilution in TBS-T for 1 h at room temperature. Blots were again washed three times with TBS-T. Protein expression was visualized using Supersignal-PLUS West Pico Solution (Thermo Scientific, Rockford, IL) according to manufacturer’s instruction. Signal was imaged using a Fluorchem SP digital imager (Alpha Innotech, San Leandro, CA). Densitometry analysis comparing each protein of interest relative to β-Actin was performed using Fluorchem SP Software (Alpha Innotech, San Leandro, CA).

### Quantitation of thiamine and thiamine phosphorylates

The effect of treatment on intracellular thiamine, TMP, and TPP was established using ion-paired reversed phase high-performance liquid chromatography (HPLC) as previously described [55]. Media was aspirated from treatment dishes and cells were trypsinized at 37° C. Cells were collected, rinsed with an equal volume of ice-cold TD 1640 medium and pelleted by centrifugation at 500 × g for 5 min at 4° C in an Allegra X-22R centrifuge (Beckman Coulter, Brea, CA). The supernatant was aspirated and cells were subsequently washed an additional two times with ice-cold PBS. If not immediately used for extraction and analysis via HPLC, pellets were stored at –80° C. Precipitated protein pellets isolated from extraction process were solubilized with 100 mM NaOH. Protein concentration was determined using a BCA protein quantification kit (Thermo Scientific, Rockford, IL) according to manufacturer’s protocol. The total thiamine/thiamine phosphate metabolite level (pmol) determined by HPLC was then normalized to total protein (mg).

### Determination of TPK1 enzymatic activity

The functionality of TPK1 was determined by an *ex vitro* enzymatic assay [56]. TPK1 catalyzes the transfer of two phosphate groups onto thiamine in the presence of adenosine triphosphate (ATP) and Mg^2+^. Cell lysates were prepared by washing treated cells with ice-cold PBS followed by lysis using mammalian protein extraction reagent (M-PER, Thermo Scientific, Rockford, IL) containing 10 μL/mL of EDTA free-100X Mammalian Protease Arrest (G-Biosciences, St. Louis, MO). Lysates were collected and centrifuged at 17,000 × g using a Microfuge 22R Centrifuge (Beckman Coulter, Brea, CA) for 10 min at 4° C to pellet cellular debris. Protein concentration of the resulting supernatant were quantitated by BCA protein assay (Thermo Scientific, Rockford, IL). Lysates (0.5 mg total protein) were then combined with ATP (5 mM, disodium salt, Cayman Chemical, Ann Arbor, MI) and MgSO_4_ (10 mM) in 0.02 M Tris-HCl reaction buffer (pH8.6). Thiamine (10 μM) was added to initiate the TPK1 reaction, which was allowed to proceed at 37° C for 30 min. To stop the reaction, proteins were precipitated by the addition of 10% trichloroacetic acid and the mixture was immediately placed on ice. The extent of TPP produced was determined via HPLC as described above.

### Determination of TKT enzymatic activity

Methodology to determine TKT enzymatic activity was adapted from Chamberlain *et al.* [57]. This assay determines the functionality of TKT protein present in lysate samples based on the proportional reduction of NADH over time. In the clinic, thiamine deficiency can be diagnosed based on the changes of TKT activity with the addition of exogenous TPP using this assay [57]. Cell lysates were prepared by washing treated cells with ice-cold PBS followed by lysis using mammalian protein extraction reagent (M-PER, Thermo Scientific, Rockford, IL) containing 10 μL/mL of EDTA free-100X Mammalian Protease Arrest (G-Biosciences, St. Louis, MO). Lysates were collected and centrifuged at 17,000xg using a Microfuge 22R Centrifuge (Beckman Coulter, Brea, CA) for 10 min at 4° C. Protein concentration of the resulting supernatant were quantitated by BCA protein assay (Thermo Scientific, Rockford, IL). 50 μg of total protein was used in a 250 μL reaction mixture with 100 mM Tris-HCl (pH 8.0), 15 mM ribose-5-phosphate, 200 μU/μL α-glycerophosphate dehydrogenase, 2.5 mU/μLtriosephosphate-isomerase, and 250 μM β-nicotinamideadeninedinucleotide reduced sodium salt (NADH). The reaction mixture was incubated at 37° C and the change in absorbance (340 nm) was recorded for 120 min at 15 min increments and normalized to total protein content.

### Determination of intracellular ROS levels

Intracellular levels of ROS were determined by the general oxidative stress indicator CM-H2DCFDA. For hypoxic quantification, the mitochondrial targeted probe MitoSOX was also used (Invitogen, Eugene, OR). Following treatments, plated cells were washed with 37° C PBS and then loaded with either cell permeable fluorescent probe dissolved in Hank’s Buffered Salt Solution (HBSS) at a concentration of 5 μM for 30 min at 37° C. Cells were subsequently washed with PBS to remove any remaining probe and then trypsinized for 3 min at 37° C. Cells were collected, washed with ice-cold PBS, then suspended in HBSS with 1 μg/mL propidium iodide (PI). Sample fluorescence was determined by flow cytometry using the FL1 and FL3 channels of a CyAn ADP analyzer (Bechman Coulter, Brea CA). Data were analyzed using FlowJo v.10 software (FlowJO, LLC, Ashland, OR).

### Quantitation of cellular proliferation

Cellular proliferation was determined by cell counting with trypan blue exclusion. Media from treatment dishes was aspirated and cells were trypsinized for 3 min at 37° C. An equal volume of cold RPMI 1640 was added to neutralize trypsin. Live cell count was determined using a 1:1 dilution with trypan blue using a TC-20 automated cell counter (BioRad, Hercules, CA). Crystal violet staining was utilized as a visual representation for the changes in proliferation. Adherent cells were fixed in buffered formalin at room temperature for 30 min. Formalin was removed and replaced with 0.5% crystal violet for 10 min at room temperature. Crystal violet was then removed and the culture dishes were washed three times with deionized water and allowed to dry prior to imaging.

### Statistical analysis

All experiments were performed with a minimum of three independent replicates. Depending on the data set, statistical significance (*p* < 0.05) was established using either an unpaired student’s *T*-test or a one-way analysis of variance (ANOVA) with Tukey’s post hoc test using GraphPad Prism 6^®^ (GraphPad Software, La Jolla, CA).

## SUPPLEMENTARY MATERIALS FIGURES AND TABLES



## Abbreviations

Thiamine: Vitamin B1
ROS: Reactive Oxygen Species
NFE2L2: Nuclear Factor Erythroid-2–Related Factor 2
HIF-1α: Hypoxia-Inducible Factor-1α
PDK1: Pyruvate Dehydrogenase Kinase-1
PDHA1: Pyruvate Dehydrogenase
TPP: Thiamine Pyrophosphate
THTR1: Thiamine Transporter 1
THTR2: Thiamine Transporter 2
TPK1: Thiamine Pyrophosphokinase-1
OGDH: α-Ketoglutarate Dehydrogenase
TKT: Transketolase
PPP: Pentose Phosphate Pathway
DMOG: Dimethyloxalylglycine
HIF-1α CA: Constitutively Active Hypoxia-Inducible Factor-1α
NAC: N-acetylcysteine
ASC: Ascorbate
DOX: Doxorubicin
CDDP: Cisplatin
AA: Antimycin A
TBHP: *tert*-Butyl Hydroperoxide
NaF: Sodium Fluoride
MTX: Methotrexate
TMP: Thiamine Monophosphate
TRLX: Trolox
MitoQ: MitoQuinone
RDA: Recommended Daily Allowance
FBS: Fetal Bovine Serum
TD 1640: Thiamine-deficient RPMI 1640
