# Supplementary material for: The adaptive regulation of thiamine pyrophosphokinase-1 facilitates malignant growth during supplemental thiamine conditions
Source: Oncotarget. 2018 Oct 23;9(83):35422–38. doi: 10.18632/oncotarget.26259 (PMC6226039; doi:10.18632/oncotarget.26259)
Supplement: Supplementary file 1 [file oncotarget-09-35422-s001.pdf]

The adaptive regulation of thiamine pyrophosphokinase-1 facilitates malignant growth during supplemental thiamine conditions

SUPPLEMENTARY MATERIALS

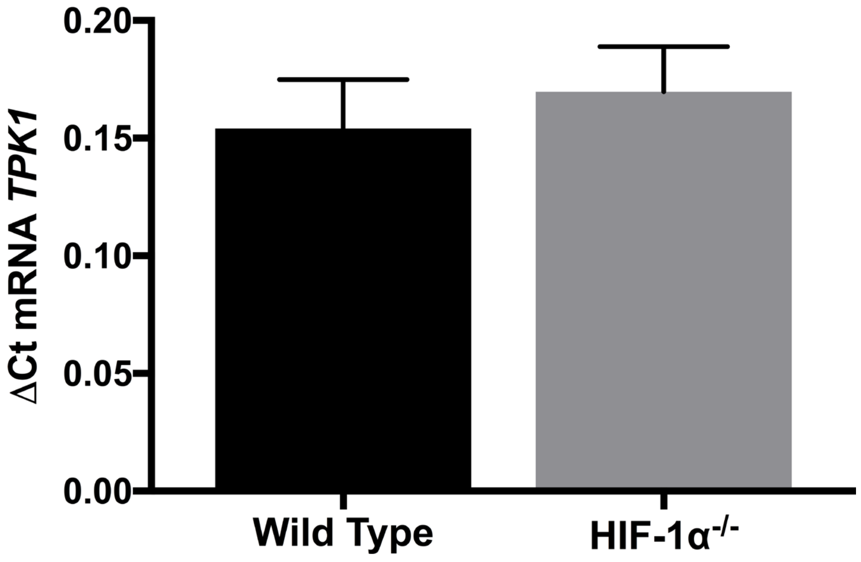

Supplementary Figure 1: mRNA expression of TPK1 in wild type and HIF-1α$^{-/-}$ HCT 116 cells. Relative mRNA expression level of *TPK1* determined by qRT-PCR analysis in wild type and HIF-1α$^{-/-}$ HCT 116 cells seeded at 1250 cells/cm^2 and cultured for 96 h normalized by the $2^{-\Delta Ct}$ method.

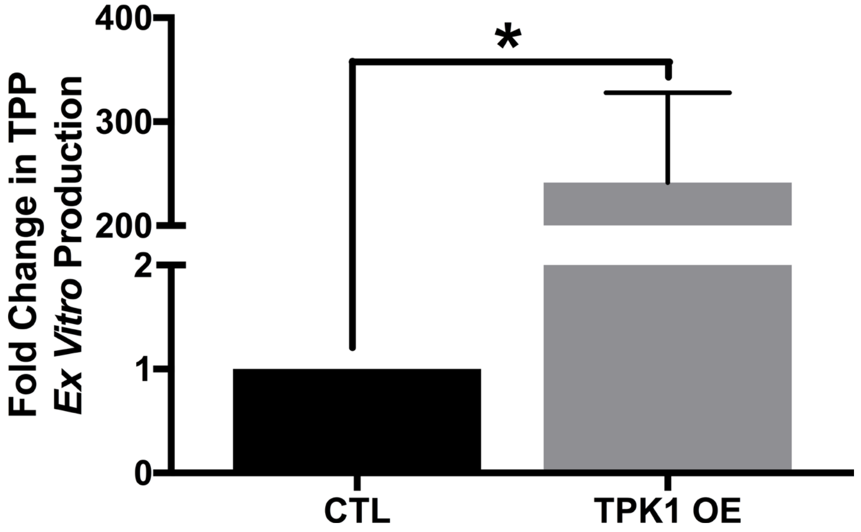

Supplementary Figure 2: *Ex Vitro* functionality of TPK1 overexpression in HCT 116 cells. HPLC analysis demonstrating *ex vitro* TPP production as fold change in TPP ± SD established in lysates isolated from wild type HCT 116 transfected with pcDNA-*TPK1* (TPK1 OE) vector for 72 h relative to control (CTL) cells. (*) Represents statistically significant difference ($p < 0.05$) based on results of an unpaired student's *t*-test.

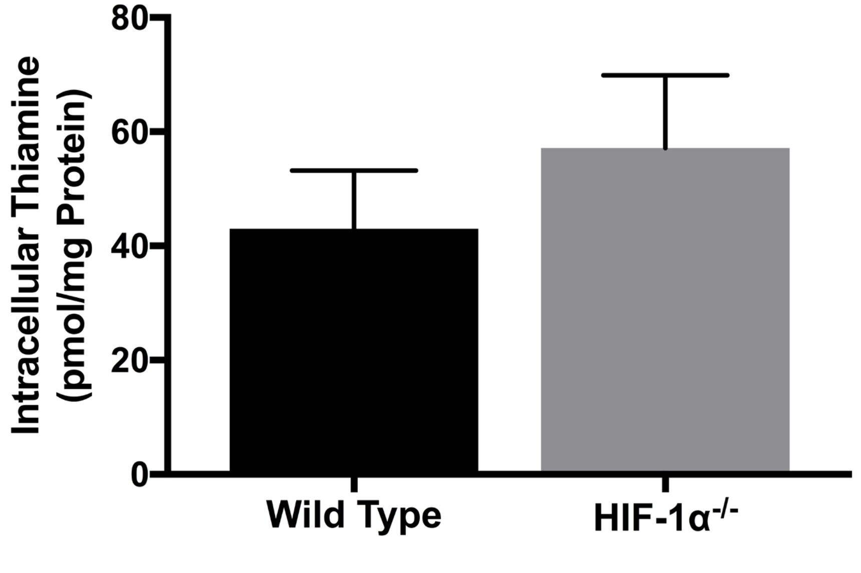

Supplementary Figure 3: Thiamine levels in wild type and HIF-1$\alpha^{-/-}$ HCT 116 cells. HPLC analysis demonstrating intracellular thiamine level ± SD established in wild type and HIF-1$\alpha^{-/-}$ HCT 116 cells seeded at 1250 cells/cm^2 and cultured for 96 h.

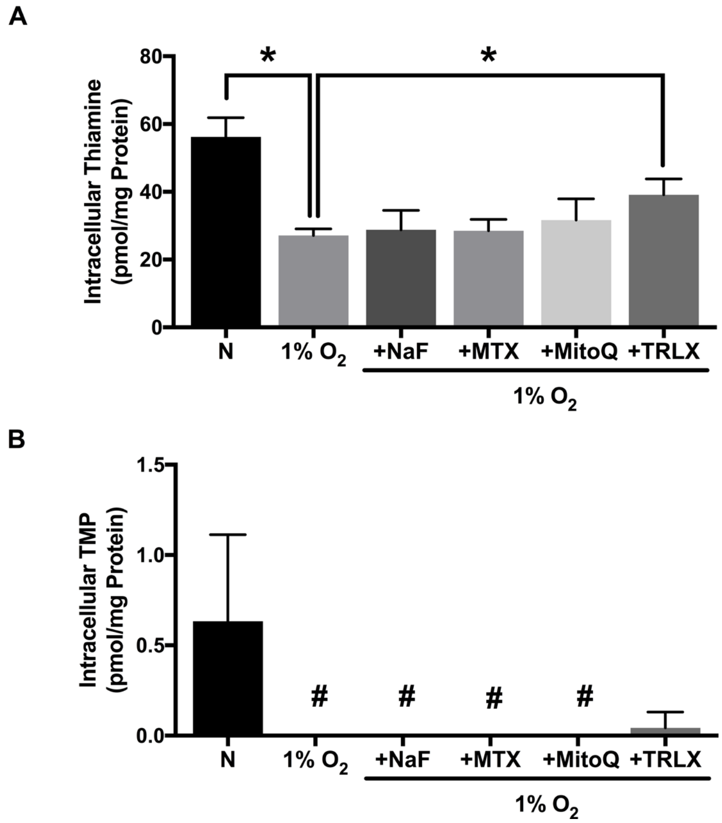

Supplementary Figure 4: Thiamine and TMP levels in HCT 116 cells treated with NaF, MTX, TRLX and MitoQ. HPLC analysis demonstrating intracellular (**A**) thiamine and (**B**) TMP levels ± SD established in wild type HCT 116 cells seeded at 25,000 cells/cm^2 and pretreated with 500 µM NaF, 100 µM MTX, 500 µM TRLX or 10 µM MitoQ for 12 h prior to hypoxic exposure for 24 h with sustained exposure to each compound. (*) Represents statistically significant difference ($p < 0.05$) based on results of an unpaired student's *t*-test. (#) Represents concentration result below the limits of quantification for assay design.

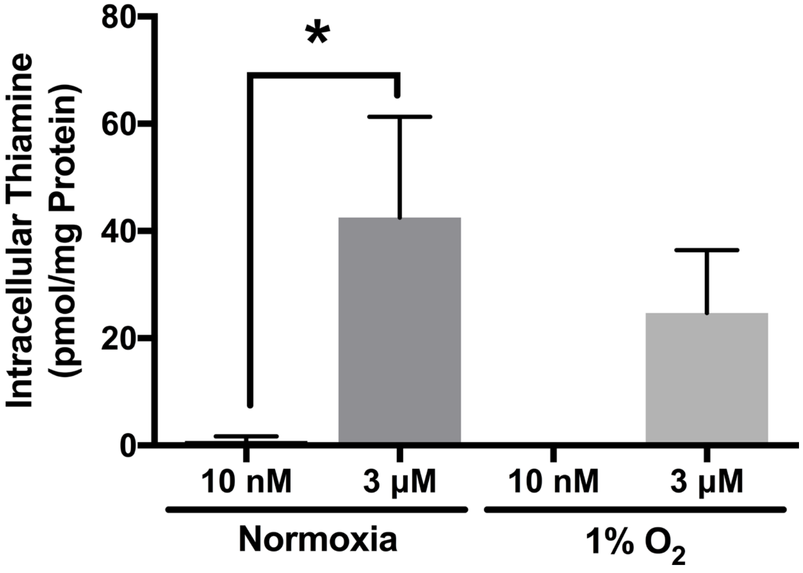

Supplementary Figure 5: Thiamine levels in wild type HCT 116 cells grown under physiological and supplemental thiamine conditions. HPLC analysis demonstrating intracellular thiamine levels ± SD established in wild type HCT 116 cells exposed to 10 nM or 3 µM thiamine for 5 d prior to seeding at 50,000 cells/cm^2 and exposure to 1% O_2 for 24 h. (*) Represents statistically significant difference ($p < 0.05$) based on results of one-way ANOVA with Tukey's post-hoc test for multiple comparisons.

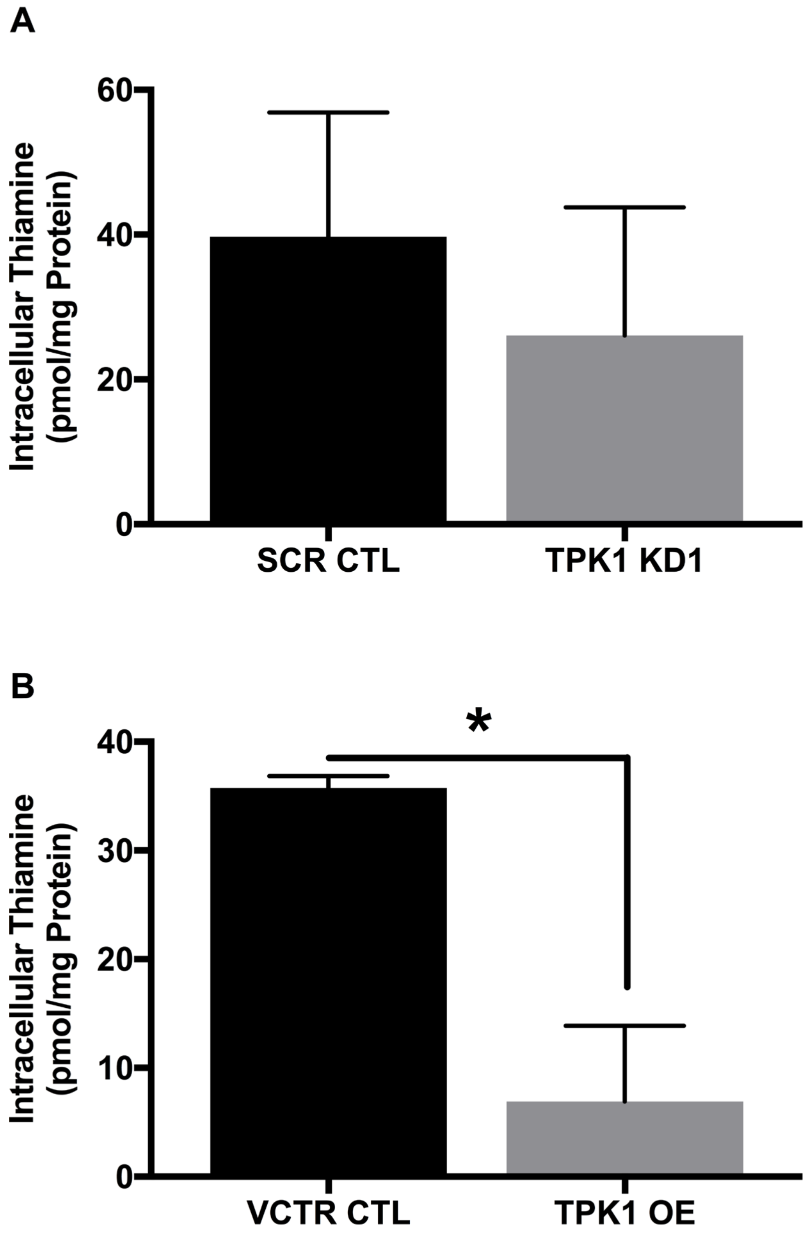

Supplementary Figure 6: Thiamine levels following TPK1 knockdown and overexpression in HCT 116 cells. HPLC analysis demonstrating mean intracellular thiamine levels ± SD established in wild type HCT 116 cells transfected with (**A**) SCR CTL and TPK1 KD1 siRNA or (**B**) VCTR CTL and TPK1 OE vectors for 72 h. (*) Represents statistically significant difference ($p < 0.05$) based on results of an unpaired student's *t*-test.

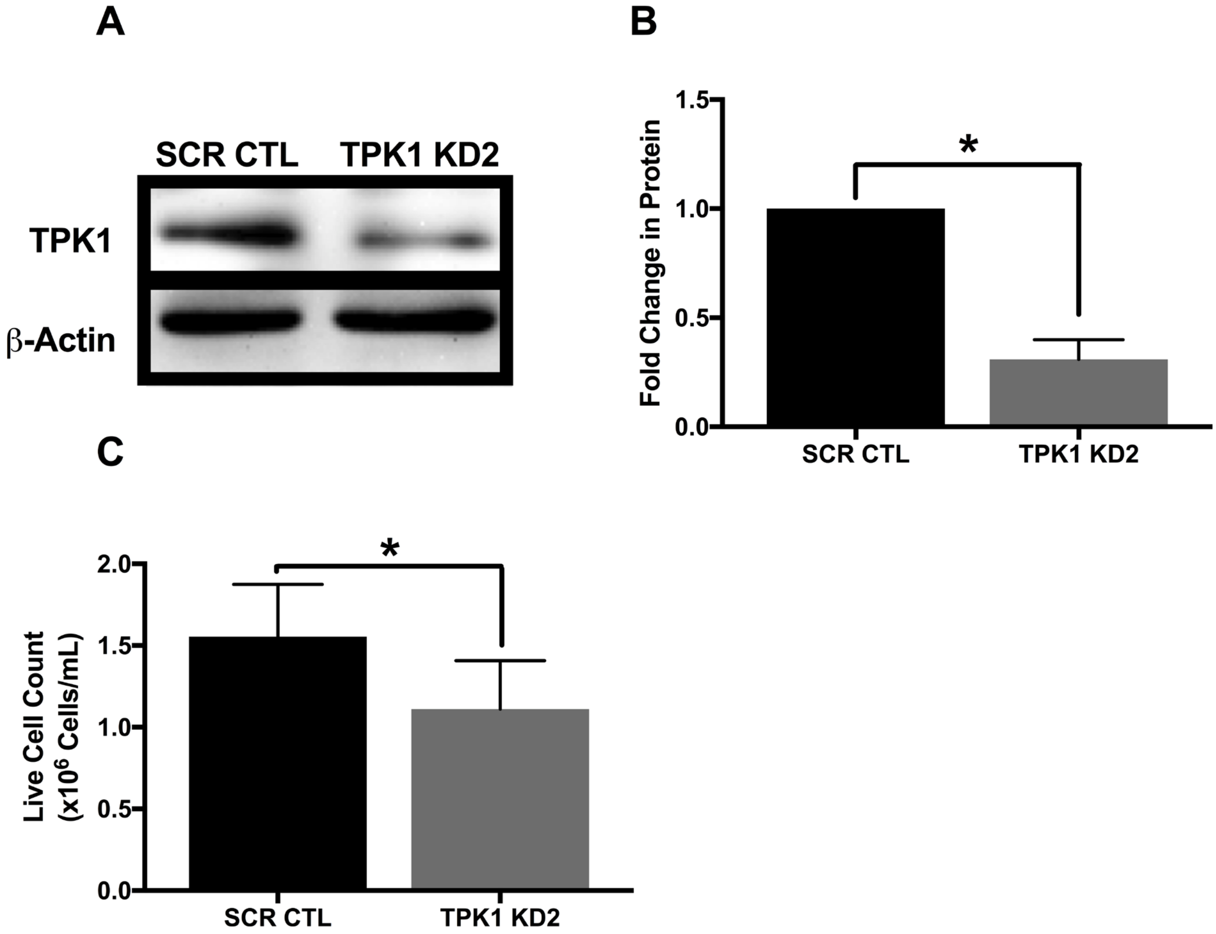

Supplementary Figure 7: Validation of TPK1 knockdown and growth effects using alternative siRNA construct. (**A**) Representative Western blot demonstrating TPK1 expression in WCLs isolated from wild type HCT 116 cells transfected with non-silencing scramble control (SCR CTL) or TPK1 targeted (TPK1 KD2) siRNA for 72 h. (**B**) Densitometry analysis of fold change in TPK1 expression ± SD in wildtype HCT 116 cells transfected with TPK1 KD2 compared to SCR CTL siRNA for 72 h. (**C**) Effect of TPK1 knockdown on tumor cell proliferation demonstrated by mean live cell count ± SD determined by trypan blue exclusion for wild type HCT 116 cells transfected with SCR CTL or TPK1 KD2 siRNA for 96 h. (*) Represents statistically significant difference ($p < 0.05$) based on results of an unpaired student's *t*-test.

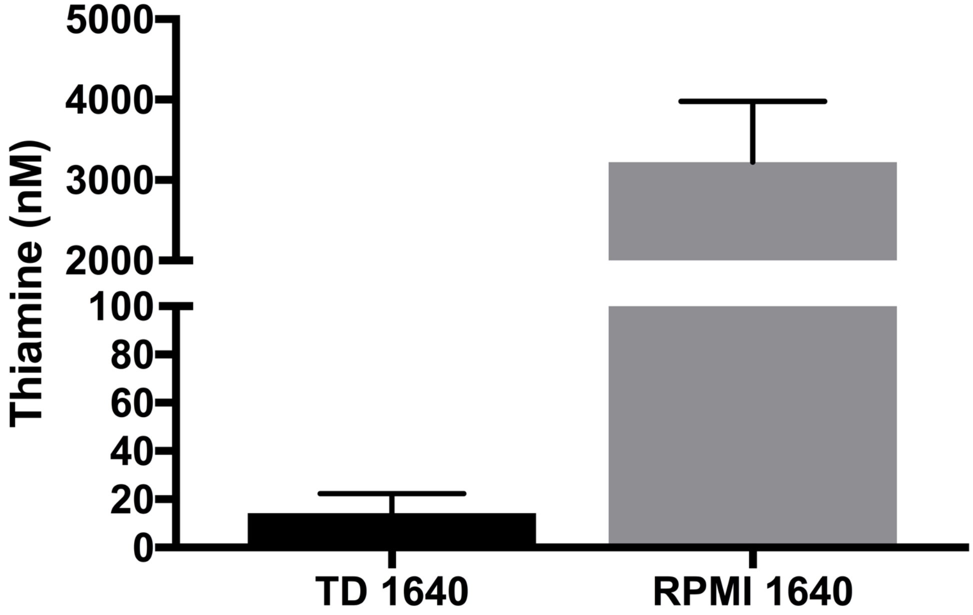

Supplementary Figure 8: Thiamine concentration of cell culture medium. HPLC analysis demonstrating thiamine concentration ± SD established in TD 1640 and RPMI 1640 following supplementation with 10% fetal bovine serum (FBS) to prepare complete growth medium.

Supplementary Table 1: Research Registry Identification (RRID) for cell lines

Cell line	ATCC catalog number	RRID
MCF7	HTB-22	CVCL_0031
MDA-MB-231	CRM-HTB-26	CVCL_0062
LN-18	CRL-2610	CVCL_0392
U-87 MG	HTB-14	CVCL_0022
Caco-2	HTB-37	CVCL_0025
HCT 116	CCL-247	CVCL_0291
Hutu 80	HTB-40	CVCL_1301

Supplementary Table 2: siRNA constructs used to mediate TPK1 knockdown

Construct	Sense and antisense sequences	Target sequence
Hs_TPK1_5<br>TPK1 KD1	5′-ccuggugcaucgaaauguatt-3′(sense)<br>5′-uacauuucgaugcaccaggtt-3′ (antisense)	5′-aacctggtgcatcgaaatg-3′
Hs_TPK1_6<br>TPK1 KD2	5′-gguugucuguagagaaugatt-3′ (sense)<br>5′-ucauucucuacagacaacctg-3′(antisense)	5′-caggttgtctgtagagaatga-3′

Supplemental Table 3: Primer sequences and roche universalprobe library probe pairs for real time-PCR analysis

Gene	Forward and reverse primer sequences	Probe
TBP	F: 5′-cggctgtttaacttcgcttc-3′<br>R: 5′-cacacgccaagaaacagtga-3′	3
TPK1	F: 5′-gcctttaccccgttggag-3′<br>R: 5′-ccaaaggctgattaagaattacaag-3′	48
LDHA	F: 5′-gtccttggggaacatggag-3′<br>R: 5′-ttcagagagacaccagcaaca-3′	47
VEGF	F: 5′-cagactcgcgttgcaaga-3′<br>R: 5′-gagagatctggttcccgaaa-3′	12
SLC2A1	F: 5′-gcccatgtatgtgggtgaa-3′<br>R: 5′-agtccaggccgaacacct-3′	81

Supplementary Table 4: Primary antibodies used for Western blot analysis

Protein of interest (Predicted MW)	Manufacturer (Catalog number)	Dilution	Secondary antibody	Antibody registry ID
TPK1 (27kDa)	Genetex (GTX103943)	1:500	Goat Anti-Rabbit	AB_1952355
LDHA (37kDa)	Genetex (GTX101416)	1:1000	Goat Anti-Rabbit	AB_10726413
b-Actin (42kDa)	Sigma-Aldrich (A2228)	1:1000	Goat Anti-Mouse	AB_476697
HIF-1a (93kDa)	Genetex (GTX127309)	1:1000	Goat Anti-Rabbit	AB_2616089
